# Co‐production of biofuel, bioplastics and biochemicals during extended fermentation of *Halomonas bluephagenesis*

**DOI:** 10.1111/1751-7915.14158

**Published:** 2022-11-09

**Authors:** Helen Park, Helen S. Toogood, Guo‐Qiang Chen, Nigel S. Scrutton

**Affiliations:** ^1^ EPSRC/BBSRC Future Biomanufacturing Research Hub, EPSRC Synthetic Biology Research Centre SYNBIOCHEM Manchester Institute of Biotechnology and School of Chemistry The University of Manchester Manchester UK; ^2^ Center for Synthetic and Systems Biology, School of Life Sciences, Tsinghua‐Peking Center for Life Sciences Tsinghua University Beijing China

## Abstract

*Halomonas bluephagenesis* TD1.0 was engineered to produce the biofuel propane, bioplastic poly‐3‐hydroxybutyrate (PHB), and biochemicals mandelate and hydroxymandelate in a single, semi‐continuous batch fermentation under non‐sterile conditions. Multi‐product separation was achieved by segregation of the headspace gas (propane), fermentation broth ([hydroxy]mandelate) and cellular biomass (PHB). Engineering was performed by incorporating the genes encoding fatty acid photodecarboxylase (CvFAP) and hydroxymandelic acid synthase (SyHMAS) into a *H. bluephagenesis hmgCAB* cassette knockout to channel flux towards (hydroxy)mandelate. Design of Experiment strategies were coupled with fermentation trials to simultaneously optimize each product. Propane and mandelate titres were the highest reported for *H. bluephagenesis* (62 g/gDCW and 71 ± 10 mg/L respectively) with PHB titres (69% g/gDCW) comparable to other published studies. This proof‐of‐concept achievement of four easily separated products within one fermentation is a novel achievement probing the versatility of biotechnology, further elevating *H. bluephagenesis* as a Next Generation Industrial Biotechnology (NGIB) chassis by producing highly valued products at a reduced cost.

## Graphical abstract

*Halomonas bluephagenesis* TD1.0 was engineered to generate multiple products propane, poly‐3‐hydroxybutyrate and (hydroxy)mandate. These compounds are easily purified due to their location in the gas, phase, cell pellet and culture supernatant, respectively. This proof of principle study shows the potential application of multi‐product biosynthesis within an industrially relevant host as a route to renewable and sustainable bio manufacturing.

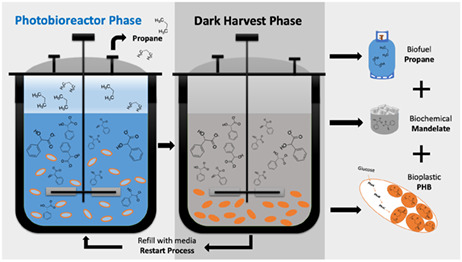

## INTRODUCTION

A major global challenge of the 21st century is to mitigate against climate change by reducing our production of greenhouse gases and reliance on fossil fuels (Sanz‐Hernández et al., 2019). Global initiatives call for greenhouse gas emission restrictions, waste biomaterial recycling, and for renewable and sustainable alternative feedstocks and biomaterials. Synthetic biology, through microbial and fermentation engineering, provides an attractive solution to achieve a circular economy with near or net zero energy and materials production. Therefore, sustainable routes towards bioenergy, biomaterials and fine biochemicals are currently the focus of intense study with the aim of generating cost‐effective industrial‐scaled processes (Amer, Hoeven, et al., 2020; Amer, Wojcik, et al., 2020).

The synthetic biology revolution has accelerated the development of ‘green’ routes to biologically derived chemicals and fuels by engineering microorganisms with naturally occurring or de novo enzymatic pathways (Clarke & Kitney, 2020). However, successful commercialization of biotechnological routes relies on its cost competitiveness with existing synthetic natural supply routes. A major bottleneck to attaining commercially viable synthetic biology‐based processes is the cost of microbial fermentations, which often require expensive and specialized raw materials, high energy and fresh water consumption in non‐continuous processes, and difficult downstream separation procedures (Chen & Jiang, 2018). Significant reductions in fermentation process costs could be achieved by transitioning microbial chassis to the robust halophilic industrial host *Halomonas bluephagenesis* (Zhang et al., 2018), which is capable of growing under non‐sterile conditions in seawater on waste biomaterials without microbial contamination (Tao et al., 2017). When cultivated under high salinity (20–150 g/L NaCl; Chen & Jiang, 2018) and alkaline pH (9–11), *H*. *bluephagenesis* was shown to grow continuously at an industrial‐scale (>1000 tonnes) for over 3 years with no decline in growth potential (Tao et al., 2017). The ever‐expanding *Halomonas* genetic toolbox includes the development of novel inducible and constitutive expression systems (Zhao et al., 2017) and stable genome integration strategies (Amer, Wojcik, et al., 2020; Chen et al., 2017), enabling a variety of non‐native secondary products to be generated (Amer, Wojcik, et al., 2020; Fu et al., 2014; Li et al., 2017).

A secondary approach to realizing commercially successful biotechnological applications is through the co‐expression of multiple products from the same bioprocess, with at least one of high economic value. This approach has been attempted previously in *Halomonas* and other organisms with studies describing the co‐production of polyhydroxyalkanoates (PHA; Liang & Qi, 2014) or algal biomass (Chandra et al., 2019; 't Lam et al., 2018) with ectoine, amino acids, biosurfactants, organic acids, pigments and proteins (Li et al., 2017; Ma et al., 2020; Yadav et al., 2021). Commercially relevant post‐fermentation downstream processing can be more efficiently achieved if the target co‐products were sequestered within separate phases of the fermentation, allowing for simple purification from each other. For example, *Halomonas* naturally produces PHA, which is deposited within the cellular biomass (Chen et al., 2017). Co‐expression with (hydroxy)mandelate (secreted into the aqueous supernatant; Li, Zhao, et al., 2016) and bio‐propane (eliminated into the headspace gas; Kallio et al., 2014) would achieve multi‐product biosynthesis of products that can be purified from each other using simple techniques.

**FIGURE 1:**
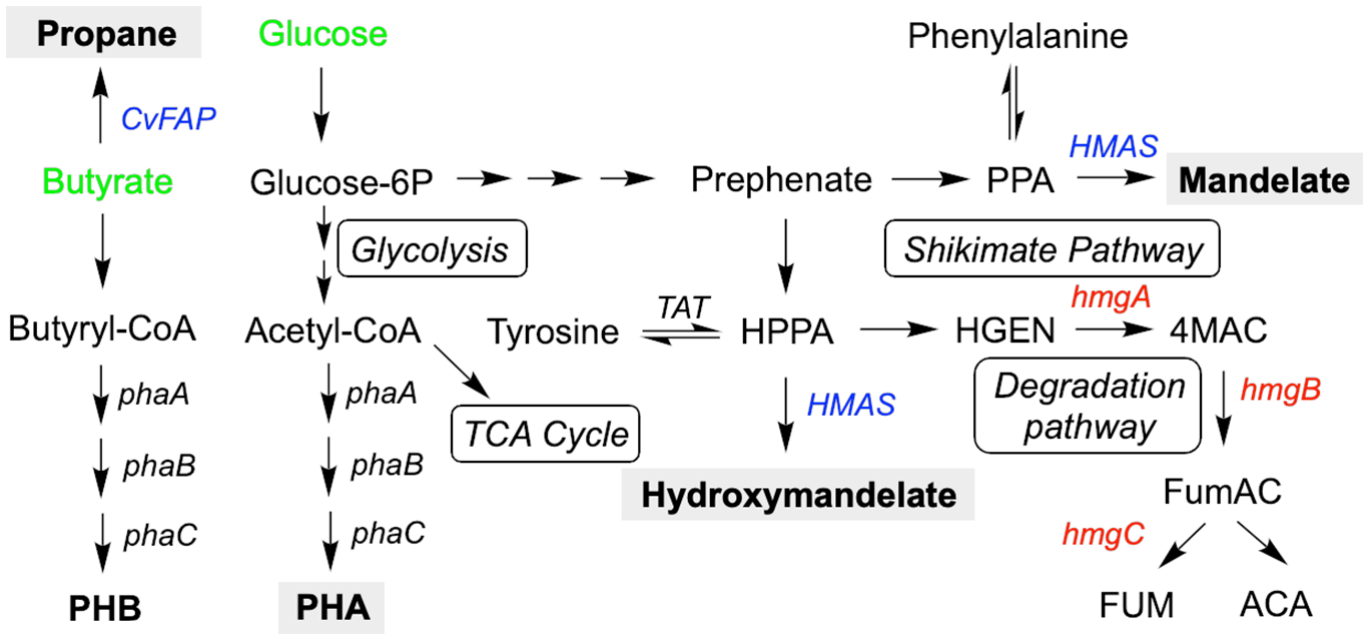
Schematic view of the recombinant *Halomonas bluephagenesis* pathways to PHA, propane, (hydroxy)mandelate and the degradation pathway of tyrosine. Enzymes incorporated into *H. bluephagenesis* TD1.0 are shows in blue, while red enzymes are targets for knock outs. Green compounds are externally fed to *H. bluephagenesis*. Metabolites: ACA = acetoacetate; Fum = fumarate; FumAC = fumarylacetoacetate; glucose‐6P = glucose‐6‐phosphate; HGEN = homogentisate; HPPA = 4‐hydroxy‐phenylpyruvic acid; PPA = 4‐phenylpyruvate; PHA = polyhydroxyalkanoate; PHB = polyhydroxybutyrate; 4MAC = maleylacetoacetate. Enzymes: CvFAP = fatty acid photodecarboxylase; HMAS = hydroxymendelic acid synthase; phaA = 3‐ketothiolase; phaB = acetoacetyl‐CoA reductase; phaC = PHB synthase; hmgA = homogentisate 1,2‐dioxygenase; hmgB = 4‐maleylacetoacetate isomerase; hmgC = fumarylacetoacetate hydrolase; TAT = tyrosine aminotransferase.

Polyhydroxyalkanoates are insoluble carbon storage polymers that reversibly accumulates under conditions of nitrogen limitation and high oxygenation, with glucose being the most favourable carbon source (Johnson et al., 2010). It is a biodegradable plastic (Figure 1) that is useful as a flexible material in the production of fine chemicals (drug carriers, antibiotics and vitamins), biomedical materials (vascular grafts, scaffolds and patches) and in tissue engineering (Adeleye et al., 2020; Park et al., 2005; Ye, Huang, et al., 2018). Pilot scale studies of engineered *H. bluephagenesis* in a stable continuous process at a 1‐ to 5‐m^3^ scale yielded a P(3HB‐co‐4HB) content of 74% (Ye, Huang, et al., 2018).

Hydroxymandelate is a water‐soluble compound used commercially for making compounds such as polystyrene‐like thermoplastics (Li, Zhao, et al., 2016), pharmaceutical precursors, and for use in skin care products (Debowska et al., 2015). The related compound mandelate is widely used in the production of cephalosporin antibiotics and other pharmaceuticals, and in the resolution of racemic alcohols and amines (Robinson et al., 2020; Sun et al., 2011). Both are currently derived from petroleum but can be enzymatically prepared via the shikimate pathway from the amino acids' tyrosine and phenylalanine respectively. Successful microbial production and secretion of hydroxymandelic (15.8 g/L) and mandelic acid (up to 0.97 g/L) was recently described in *Escherichia coli* (Li, Zhao, et al., 2016; Robinson et al., 2020; Sun et al., 2011) using the hydroxymandelic acid synthase enzyme from *Streptomyces yokosukanensis* (SyHMAS). However, to date (hydroxy)mandelate production in *Halomonas* sp. has not been demonstrated (Figure 1).

The final product is gaseous bio‐propane; the third most widely used fuel for domestic and transportation applications, with a global demand estimated to be around 300 million tonnes per annum (Johnson, 2019). It is an ideal biofuel since it can be ‘dropped‐in’ to existing fuel infrastructures, and it can be stored at low energy in a liquid state (de Jong & Jungmeier, 2015; Kallio et al., 2014). Propane production in both *E. coli* and *H. bluephagenesis* has been demonstrated using the blue‐light activated fatty acid photodecarboxylase enzyme from *Chlorella variabilis* (CvFAP; Heyes et al., 2020; up to 180 mg/g cells/day; Figure 1) or aldehyde deformylating oxygenase from *Prochlorococcus marinus* (3.4 mg/L; Kallio et al., 2014; Menon et al., 2015).

This study demonstrates the co‐production of PHA, (hydroxy)mandelate and bio‐propane within one engineered *H. bluephagenesis*. We show a hybrid approach combining the stable integration of constitutively expressed CvFAP and SyHMAS variants with ‘top‐up’ IPTG‐inducible plasmid‐borne SyHMAS to ensure significant titres of all products can be achieved. Additional strain engineering was performed for improved (hydroxy)mandelate production by knocking out genes involved in tyrosine degradation. Through media and fermentation optimization, this study aims to investigate the combinatorial effect of three plus product simultaneous production whilst minimizing the impact on cellular biomass production. The results demonstrate a feasible path forward for co‐production, which will require further scaled work with industrially optimized strains.

## EXPERIMENTAL PROCEDURES

### Strains and plasmids

All chemicals and solvents were commercially sourced and were of analytical grade or better. The organism *H. bluephagenesis* TD1.0 is an engineered variant of the TD01 strain previously isolated from the Aydingkol Lake in Xinjian, China (Tan et al., 2011). Cultivation was performed in high salt Luria broth (LB60; LB containing 60 g/L NaCl) pH 6.8 (conjugation) and 9.0 (recombinant enzyme expression). Minimal medium for *H*. *bluephagenesis* cultivation was a modified high salt MM63 medium pH 9 (2 g/L [NH_4_]_2_SO_4_, 13.6 g/L KH_2_PO_4_, 0.5 mg/L FeSO_4_·7H_2_O, 1.2 mg/L MgSO_4_ and 60 g/L NaCl) containing 10 g/L glucose.

A list of plasmids and genomic integrated *H*. *bluephagenesis* TD1.0 strains generated in this study can be found in Table S1 and Figure S1. The BglBrick series of vectors were obtained from Addgene (https://www.addgene.org; Lee et al., 2011). The plasmids pHal2‐T7‐FAP (IPTG)‐inducible CvFAP_G462I_, pSH‐CvFAP_G462I_, pSBR1Ks‐i‐SceI and the wild type, single (I219V) and double (I219V/S204V) variants of SyHMAS were constructed during previously published studies (Amer, Hoeven, et al., 2020; Amer, Wojcik, et al., 2020; Robinson et al., 2020). The inserts generated in this study for genomic integration contain a pKIKO‐derived chloramphenicol resistance gene flanked by FRT sequences (Amer, Hoeven, et al., 2020; Sabri et al., 2013). Further details on plasmid and medium composition can be found in the Supplementary Experimental section.

### Plasmid construction in *E. coli*

The construction of expression plasmids containing SyHMAS variants and/or CvFAP_G462I_ in pHal2 backbones (Table S1) were performed by PCR amplification of individual ‘parts’ (vector linearization and gene/control DNA inserts) using CloneAmp™ HiFi PCR Premix (Clontech; Raman & Martin, 2014). All oligonucleotide sequences used for plasmid construction can be found in Table S2. Each PCR product was analysed by agarose gel electrophoresis and the correct sized bands were excised and purified using the Monarch® Genomic DNA Purification Kit DNA purification kit (NEB). Plasmid assembly was performed by In‐Fusion® Cloning (Clontech), with each part containing the required 15 bp complementary overhangs for assembly in the correct order and orientation. To construct pHal2‐FAP_G462I_‐HMAS_I219V_, the SyHMAS variant was inserted downstream of CvFAP_G462I_ within the linearized plasmid pHal2‐T7‐FAP. For wild‐type and variant SyHMAS expression plasmids, pHal2‐T7‐FAP was linearized to remove the CvFAP_G462I_ gene and the SyHMAS PCR product was inserted downstream of the T7‐like promoter.

The genome integration and knock‐out constructs in the suicide vector (pSH) vector were generated using the protocols described above (Table S1), except plasmid assembly was facilitated using the NEBuilder® HiFi Gibson Assembly Cloning Kit (New England Biolabs; Birla & Chou, 2015). To generate the base FAP_G462I_‐HMAS_I219V_‐containing pSH construct, the SyHMAS single variant was inserted between the CvFAP and the chloramphenicol‐resistant (Chl^R^) genes of plasmid pSH‐CvFAP_G402I_. The selected genome integration sites were downstream of the constitutively expressed porin (Por; Li, Li, et al., 2016), iron–sulphur fumarate reductase frdB (FumR; Cecchini et al., 2002) and the universal stress protein COG0589 (COG; Tkaczuk et al., 2013). Pairs of homology arms for each of the three loci were amplified from the genomic DNA of *H. bluephagenesis* TD1.0 to act as upstream (HA1) and downstream (HA2) flanking DNA sequences around the CvFAP_G462I_‐HMAS_I219V_‐Chl^R^ cassette. NEBuilder assembly was performed between the CvFAP_G462I_‐HMAS_I219V_‐ChlR cassette, loci HA1/HA2 fragments and a ColE1 replication origin to form the three genome integration plasmids (pPor‐FAP‐HMAS_I219V_, pFumR‐FAP‐HMAS_I219V_ and pCOG‐FAP‐HMAS_I219V_). To generate the *hmgCAB* knockout plasmid pHal2‐tet‐hmgH1H2, NEBuilder assembly was performed between the tetracycline resistance (Tet^R^) gene from pBR322 (New England Biolabs), genome‐specific homology arms for *hmgCAB* and the origin of replication from pHal2. The homology arms are specific for the start of HmgC and end of HmgB from *H*. *bluephagenesis* TD1.0 genomic DNA, which is designed to replace the genomic *hmgCAB* with the Tet^R^ gene.

Following assembly, each plasmid was transformed into *E. coli* strain Stellar or NEB5α and incubated overnight on antibiotic‐selective LB agar plates. Individual colonies were picked, and small liquid cultures (10 ml) were cultivated overnight at 37°C using antibiotic‐selective LB medium. Following plasmid recovery and purification (Plasmid Purification DNA kit, ThermoFisher), each plasmid was sequenced to confirm the correct construct had been made. Sequence confirmed plasmids were conjugated into *H*. *bluephagenesis* TD1.0 for expression studies according to previously published methods (Amer, Wojcik, et al., 2020). During each cloning stage, protocols specified by the individual kit manufacturers were followed.

### Genomic integration and *hmgCAB* knockout

Genomic insertion of FAP‐HMAS_I219V_ constructs and *hmgCAB* deletion were performed via homologous recombination using a previously published suicide vector protocol (Amer, Wojcik, et al., 2020). In this methodology, the pSH‐based Por/FumR/COG‐FAP‐HMAS_I219V_ constructs or the knockout pHal2‐tet‐hmgH1H2 plasmid are co‐expressed within *H*. *bluephagenesis* TD1.0 with a second kanamycin and spectinomycin‐resistant plasmid pSBR1Ks‐i‐SceI. Further details of the genome integration protocol can be found in the Supplementary Experimental section. Successful integration of the FAP‐HMAS_I219V_ cassette was seen as growth of *H*. *bluephagenesis* on chloramphenicol‐selective LB60 medium (tetracycline for successful *hmgCAB* ^−^ knockout). Integration was confirmed by colony PCR, genomic sequencing, and in vivo propane production after pSceI plasmid curing (Fu et al., 2014; Qin et al., 2018).

### Small scale in vivo production of propane, (hydroxy)mandelate and PHB

Propane production was determined in *H*. *bluephagenesis* strains containing CvFAP_G462I_ in the genome or on a plasmid (pHal2‐FAP‐HMAS_WT or I219V_). Plasmid‐borne cultures (10 ml) were incubated in LB60 pH 6.8 containing 50 μg/ml spectinomycin at 30°C until the OD 600 nm reached 0.7–0.8. Protein expression was induced with 0.1 M IPTG, followed by the addition of 50 mM butyric acid (propane precursor). For *H*. *bluephagenesis* strains lacking a pHal2‐based plasmid, no spectinomycin or IPTG was added. Triplicate culture aliquots (1 ml) were sealed within 4 ml airtight glass vials and incubated at 30°C for 10 h at 180 rpm under a 455 nm LED blue light panel. Propane concentration was determined via manual headspace sampling and analysis using an Agilent 490 Micro GC (Amer, Wojcik, et al., 2020).

Mandelate and hydroxymandelate production was determined in *H*. *bluephagenesis* strains containing wild‐type or variant SyHMAS within a plasmid (pHal2‐FAP‐HMAS_I219V_ or pHal2‐HMAS variant) or encoded on the genome. Duplicate cultures (30 ml) were set up in LB60 pH 6.7 containing 50 μg/ml spectinomycin (plasmid‐based only) and supplemented with tyrosine (0–3 g/L), phenylalanine (0–3 g/L), glucose (0–30 g/L), phenylpyruvate (10–20 mM) and/or glycerol (0–10 g/L). The cultures were incubated at 30°C with 180 rpm agitation until the OD 600 nm reached 1.5, followed by induction with 0.1 M IPTG and a further 48 h incubation at 30°C. To determine the concentration of (hydroxy)mandelate, triplicate culture samples (900 μl) were mixed 1:1(v/v) with methanol and diluted 100‐fold in water. Samples were analysed for (hydroxy)mandelate concentration by LCMS (Robinson et al., 2020).

PHA production was determined using *H*. *bluephagenesis* constructs cultivated in antibiotic selective LB60 pH 9 containing 15 g/L glucose or modified high salt MM63 medium pH 9 containing 10 g/L glucose. Cultivations requiring glucose depletion monitoring were performed in the modified high salt MM63 medium. Cultures (300 ml) in antibiotic‐selective medium were cultivated at 30°C with 180 rpm agitation, followed by the addition of 0.1 M IPTG (plasmid‐borne constructs) and/or 1.6–3.6 g/L butyric acid (for propane co‐production studies) when the culture optical density reached 0.8–1.0. The cultivation was continued, as before, and culture aliquots (25 ml) were withdrawn periodically for PHA analysis. Culture sample cell pellets were washed twice by resuspending in 5 ml water and centrifuging at 3220 *g* for 10 min. The washed pellet was flash frozen in liquid nitrogen and lyophilized for 12 h.

### Optimization of secondary product generation using design of experiment

Optimization of (hydroxy)mandelate production was performed using a statistical (DOE) approach to evaluate multiple parameters likely to affect titres with reduced experimental effort. In this screen, all five parameters were varied, and 17 individual growth conditions were identified for comparative in vivo testing (Table S3). A second more focused screen was performed, based on the initial screen, that varied only the concentrations of glucose, tyrosine and phenylalanine (Table S4). Further details on the statistical software, models and analysis are described in the Supplementary Experimental section.

### Photobioreactor cultivation of *H. bluephagenesis*

Photobioreactor cultivation (450 ml) was performed using a thermostatic flat panel FMT 150 (Photon Systems Instruments, Czech Republic) with optical cell density monitoring (OD 680 nm), pH and temperature control and an integral LED blue light panel (465 nm; Amer, Wojcik, et al., 2020). Cultivation was performed with *H*. *bluephagenesis* Por‐FAP‐HMAS_I219V_ *hmgCAB*‐ with plasmid pHal2‐HMAS_WT_ or pHal2‐HMAS_I219V_ in fed‐batch mode in LB60 pH 6.8 containing 50 μg/ml spectinomycin and 0.5 ml/L antifoam. The apparatus was pre‐equilibrated at 30°C with a 60% stirring output, constant pH control and airflow maintained at 0.3–1.25 L/min, dependent on the experiment. An overnight starter culture of *H*. *bluephagenesis* (5–10 ml) was added to achieve an initial OD 680 nm of ~0.2. The culture was maintained under ambient room light until the OD 680 nm reached 0.8–10, dependent on the experiment, followed by the addition of 0.1 M IPTG and 50 mM butyrate. One hour later, the blue LED panel was switched on (300–800 μmol photons/m^2^/s or μE) for cultures monitored for propane production. Illumination strategies varied from constant blue light to frequent on/off cycles, dependent on the experiment. Manual feeding of butyric acid to 45 mM was performed daily when propane titres began to drop. For fermentations with constant feeding regimes, an automatic timer pumped ~8 ml of LB60 medium containing 2.5 M sodium butyrate, 4 M glucose and 0.15 M phenylalanine every 3 h after the blue light was switched on. To mimic an extended fed‐batch fermentation, one fermentation included periodic harvesting of 250 ml of culture and refilling with LB60 pH 6.8, followed by dark cultivation for 12 h to increase the cell density. Butyric acid (18 mmol) was added to the culture and the blue light was switched back on until the propane titre had significantly fallen. The remaining fermentation parameters were maintained until the end of the cultivation (65–100 h).

Automated fermentation headspace gas monitoring was performed to quantify propane production using a Micro GC (Amer, Wojcik, et al., 2020) at 20‐min intervals after the blue light was switched on. Culture sampling (25 ml) was performed regularly, enabling the offline quantitation of PHA and (hydroxy)mandelate using methods described above. PHA samples were withdrawn at the start of a dark phase of growth and 3–4 h after a glucose feed of 20 g/L. Additional offline analyses were performed by HPLC to monitor the depletion of glucose and butyrate, and the accumulation of acetate in the culture medium, as described previously (Amer, Wojcik, et al., 2020). The optical density probe data were corrected for non‐linearity above ~0.9 and converted to an apparent OD 600 nm and DCW (for propane calculations) using the calibration curves in Figure S2. In addition, periodic culture sampling was performed to monitor the OD 600 nm using a spectrophotometer.

### Analytical techniques

Recrystallization of mandelate was performed using an 85% aqueous solution (3 g in 10 ml water) contaminated with 15% hydroxymandelate (425 mg). The solution was heated to 30°C within an EasyMax 102 thermostat (Mettler Toledo) and the temperature was held for 10 min. The solution was seeded with mandelate crystals (50 mg) and the temperature was maintained for a further 10 min. Rapid cooling was performed at 0.1°C per minute until 20°C, and the temperature was maintained overnight with 500 rpm stirring. Mandelate crystals were vacuum filtered to remove the liquors, followed by freeze drying. Both the solids and liquor were analysed for mandelate and hydroxymandelate content by HPLC.

Quantitation of mandelate and hydroxymandelate was performed using an ultra‐performance liquid chromatography system (Waters Acquity UPLC H‐class) coupled to a Xevo TQ‐S triple‐quadrupole mass spectrometer (Waters Corporation, MA, USA) with an electrospray ionization source (ESI‐ mode). Compounds were separated on a Waters Acquity HSS T3 column (50 mm × 2.1 mm, 1.8 μm) using a method described previously (Robinson et al., 2020). Propane titres from small, sealed cultures and automated fermenter off gas propane detection were determined by manual headspace injection into an Agilent 490 Micro GC, containing an Al_2_O_3_/KCl column and a thermal conductivity detector (TCD; Amer, Wojcik, et al., 2020). PHA hydrolysis and methanolysis was performed by a modification of the method described previously (Ye, Hu, et al., 2018). Aqueous culture metabolites (butyrate, acetate, glucose and glycerol) were analysed by HPLC using an Agilent 1260 Infinity HPLC with a 1260 ALS autosampler, TCC SL column heater, a 1260 refractive index detector (RID). Samples were run on an Agilent Hi‐Plex H column (300 × 7.7 mm; 5 mM H_2_SO_4_) using the running conditions described previously (Amer, Hoven, et al., 2020, Amer, Wojcik, et al., 2020). In each case, sample retention times and standard curves were prepared using authentic standards. Further details of analytical methods can be found in the Supplementary Experimental section.

The relative cell viability of culture samples was determined using the CellTitre‐Glo® 2.0 kit (Promega). A modified protocol was employed where the culture was normalized to an OD 600 nm of 1. The samples were incubated with 20 μl of CellTitre‐Glo Reagent for 10 min at 37°C with 800 rpm agitation. The luminosity of quadruple aliquots (20 μl) of each sample within 384‐well white plates was measured using a BMG Labtech Clariostar plate reader. The program measured luminosity endpoint without a filter, using an emission spectrum range of 490–700.

## RESULTS

### PHA production by *H. bluephagenesis* TD1.0

**FIGURE 2:**
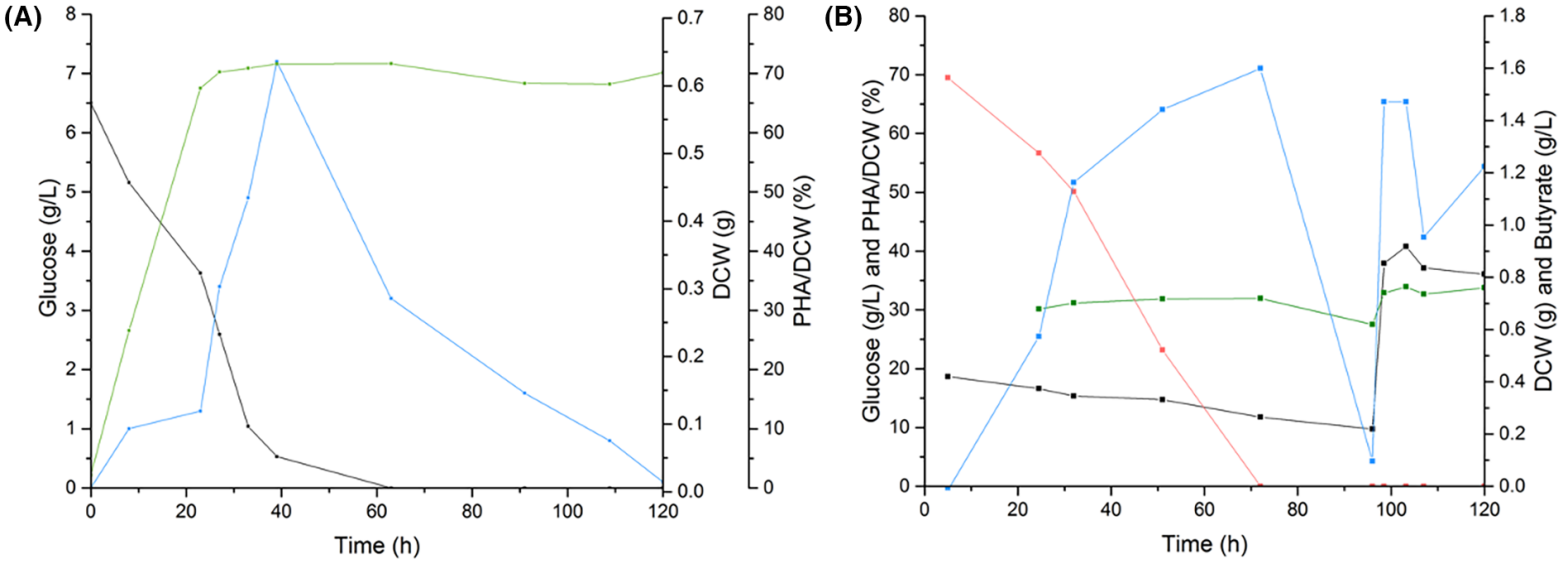
Effect of (A) glucose and (B) both glucose and butyrate levels on PHA accumulation in *Halomonas bluephagenesis* TD1.0. Cultures (300 ml) were grown in LB60 pH 6.8 containing 20 g/L glucose ±15 mM butyric acid at 30°C for 120 h. Samples were withdrawn periodically and the concentration of glucose and PHA was determined using HPLC and GC, respectively. DCW = dry cell weight. For part a, data for glucose, DCW and PHA/DCW is shown in black, green and blue, respectively. For part B, glucose, DCW, butyrate and PHA/DCW is shown in red, green, black and blue respectively.

To benchmark PHA production in non‐engineered *H. bluephagenesis* TD1.0 we generated small scale cultures in glucose enriched medium, which accumulated PHA up to 72% of cellular dry cell weight (DCW) using the native biosynthesis pathway until glucose levels had depleted to ~0.5 g/L (Figure 2A). This was followed by a rapid depolymerization of PHA, likely to provide a new carbon source for growth. This single native product strain showed comparable PHA accumulation to industrial strains of *H. bluephagenesis* (92% g/gDCW PHA) that had undergone engineering to overexpress the three PHA genes (*phaA*, *phaB* and *phaC*; Figure 1; Zhao et al., 2017).

The effect of butyrate on PHA production was investigated in the presence of glucose. This was performed as butyrate is both a propane production precursor and a secondary carbon source for *H. bluephagenesis* TD1.0 (Figure S3). Batch culture studies showing similar PHA titres were achieved with butyrate present (up to 72 g/gDCW (%)). This was accompanied by both glucose and butyrate depletion, with a rapid PHA depolymerization seen after butyric acid was completely consumed (glucose levels ~12 g/L; Figure 2B). Feeding in additional glucose at this stage resulted in a secondary spike of PHA production (66 g/gDCW (%)). This suggests the maintenance of high intracellular PHA levels is likely dependent on the concentration of a readily available carbon source. Therefore, a batch feed of high concentrations of glucose near the end of fermentation may trigger a boost on PHA production just prior to harvesting.

### (Hydroxy)mandelate production in *H. bluephagenesis* TD1.0

**FIGURE 3:**
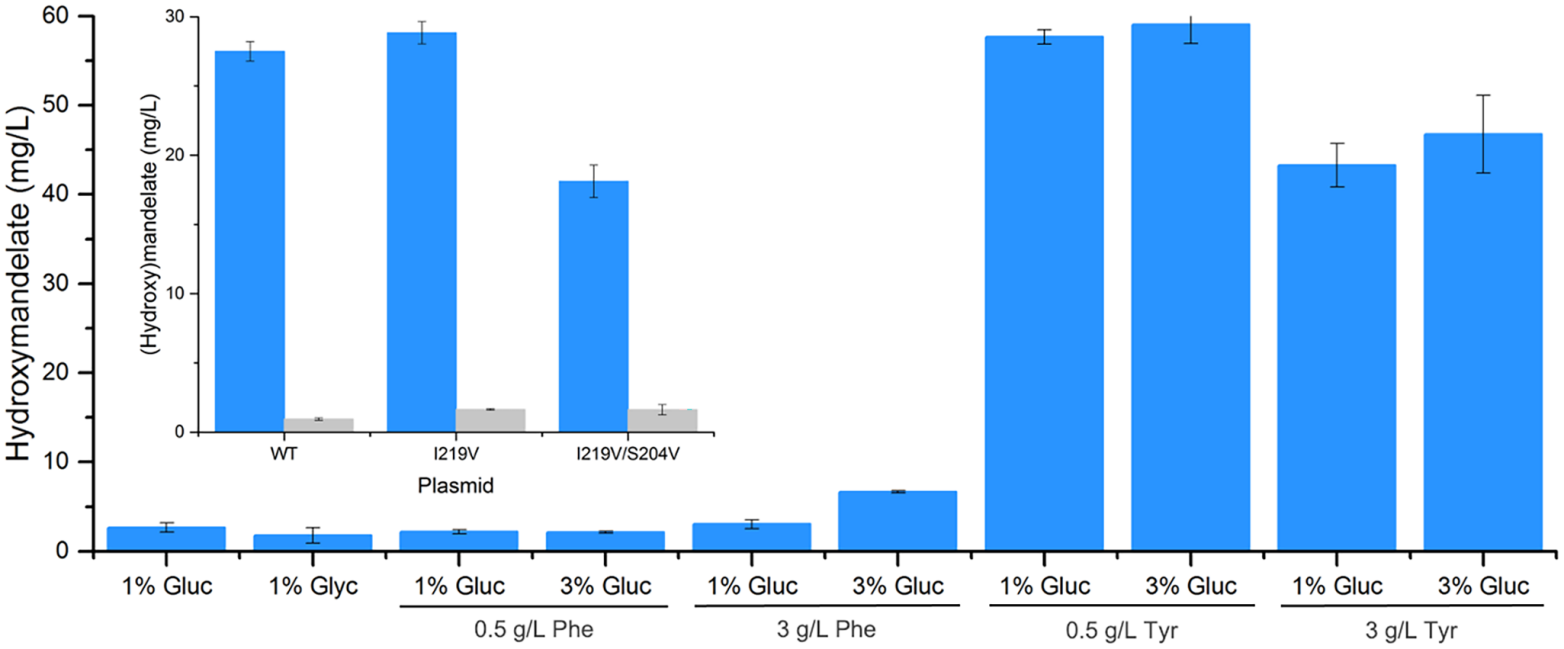
Effect of amino acid supplementation and carbon source on hydroxymandelate production in *Halomonas bluephagenesis* TD1.0 with pHal2‐HMAS_I219V_. Inset: Effect of SyHMAS variant on (hydroxy)mandelate production in the presence of 0.5 g/L tyrosine. Cultures (30 ml) were grown in LB60 pH 6.8 containing 1–3% glucose (or 1% glycerol) with or without amino acid supplementation (0.5–3 g/L Tyr or Phe). No glucose or glycerol was added to cultures in the inset. Cultures were incubated at 30°C for 48 h. (Hydroxy)mandelate concentration was determined by LCMS. Gluc = glucose; glyc = glycerol, Tyr = tyrosine, Phe = phenylalanine. Hydroxymandelate and mandelate are shown as blue and grey bars respectively.

There are no known published studies reporting (hydroxy)mandelate production in *Halomonas*. Prior studies of SyHMAS in *E. coli* generated variants I219V and S204V to increase precursor selectivity of phenylpyruvic acid (PPA) over 4‐hydroxy‐phenylpyruvic acid (HPPA), as well as improving the enantiopurity of the final product. Therefore, to benchmark (*S*)‐(hydroxy)mandelate production in *H. bluephagenesis* TD1.0, we incorporated a plasmid containing wild‐type SyHMAS and variants I219V and I219V/S204V. Cultures containing glucose or glycerol as a carbon source were supplemented with tyrosine or phenylalanine to act as precursors for hydroxymandelate and mandelate respectively. Surprisingly, *H. bluephagenesis* SyHMAS_I219V_ variant cultures showed only hydroxymandelate production (1.79–6.6 mg/L), even with phenylalanine supplementation (Figure 3). A separate screen of wild‐type and variant SyHMAS in *H. bluephagenesis* TD1.0 with tyrosine supplementation led to a significant increase in hydroxymandelate titre (43–59 mg/L) and minor mandelate production around 10‐30‐fold lower than hydroxymandelate (Figure 3 inset).

The I219V variant generated similar hydroxymandelate titres to the wild‐type construct (28.9 ± 0.8 vs. 27.5 ± 0.7 mg/L), while the SyHMAS_I219V/S204V_ variant showed a 1.6‐fold reduction in titre (18.1 ± 1.2 mg/L; Figure 3 inset). Therefore, the SyHMAS_I219V_ variant was chosen for further study, although titres of both products are significantly lower than comparable studies in *E. coli* (0.8 g/L mandelate and 4.8 g/L hydroxymandelate; Robinson et al., 2020; Sun et al., 2011). We suspect phenylalanine is acting as an alternative carbon source for *H. bluephagenesis* when glucose levels deplete (results not shown), effectively reducing the amount available to feed through to mandelate production. However, a bioinformatics search of the TD01 genome failed to find a complete phenylalanine degradation pathway or any genes annotated as phenylalanine uptake transporters (results not shown).

### Propane production in PHA‐producing *H. bluephagenesis* TD1.0

**FIGURE 4:**
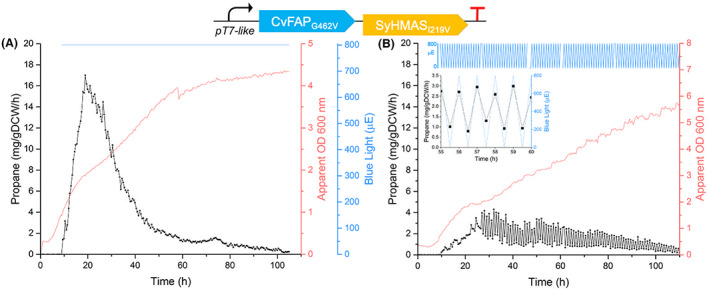
Fermentative propane production of *H. bluephagenesis* TD1.0 expressing plasmid pHal2‐FAP‐HMAS_I219V_ under (A) constant or (B) 30‐minute cycling illumination of blue light. The culture (400 ml) was grown in LB60 pH 6.8 at 30°C containing 100 mg/ml spectinomycin. IPTG induction (0.1 mM) was performed at OD 680 nm of 0.8, with the addition of 50 mM butyrate and constant blue light (800 μmol photons/m^2^/s or μE) 1 h later. For panel B, the blue light was pulsed 30 min on and 30 min off. The inset shows 6 h of the data. Cultures were maintained with constant aeration (rate) and temperature for ~100 h. online headspace gas analysis for propane production was performed using a Micro GC. Propane and culture optical density are shown as green circles and grey lines, respectively. Blue light is shown schematically by blue lines. Apparent OD 600 nm = photobioreactor optical density probe data corrected for non‐linearity using the calibration curve in Figure S16.

Propane production in *H. bluephagenesis* TD1.0 was achieved by incorporating the blue light dependent CvFAP_G462I_ gene within the SyHMAS_I219V_ plasmid. Cultivations were performed within a photobioreactor, with CvFAP_G462I_ activity switched on under illumination with either constant or cyclic (30 min on/off) blue light in the presence of butyric acid (substrate). Under constant illumination, propane production peaked around 7–10 h after induction (18.9 mg/gDCW/h), followed by a slow decline in production with culture optical density increasing (Figure 4A). This is likely due to loss of the plasmid over the extended cultivation in addition to photoinactivation of CvFAP (Heyes et al., 2020).

Extended cultivation of *H. bluephagenesis* under high intensity blue light is likely to cause cell death (Eisenstark, 1987) as well as photoinactivation of CvFAP (Lakavath et al., 2020). To understand these factors, we measured if reduced light could improve cell viability and propane production under cyclic blue light exposure, to allow *H. bluephagenesis* to replenish replicating cells and active CvFAP biosynthesis during the ‘dark’ phases. However, significantly lower propane production rates were seen than during cultivation under constant blue light (max ~4 mg/gDCW/h), likely due to half the fermentation time being spent in the dark in the recovery phase where no propane can be generated (Figure 4B). Interestingly, peak propane production rates under constant illumination were four‐fold higher than comparable studies with the related PHA‐deficient *H. bluephagenesis* TQ10 strain(~4.2 mg/gDCW/h; Amer, Wojcik, et al., 2020). These results are encouraging as they show that cumulative propane production is not significantly impacted by high levels of PHA production. Indeed, the superior fitness and propane production rate of *H. bluephagenesis* TD1.0 under blue light over a PHA‐knock out TQ10 strain suggests co‐production of PHA actually improves propane production, perhaps by acting as a UV‐light protectant (Slaninova et al., 2018).

### Genomic integration of propane and (hydroxy)mandelate production

**FIGURE 5:**
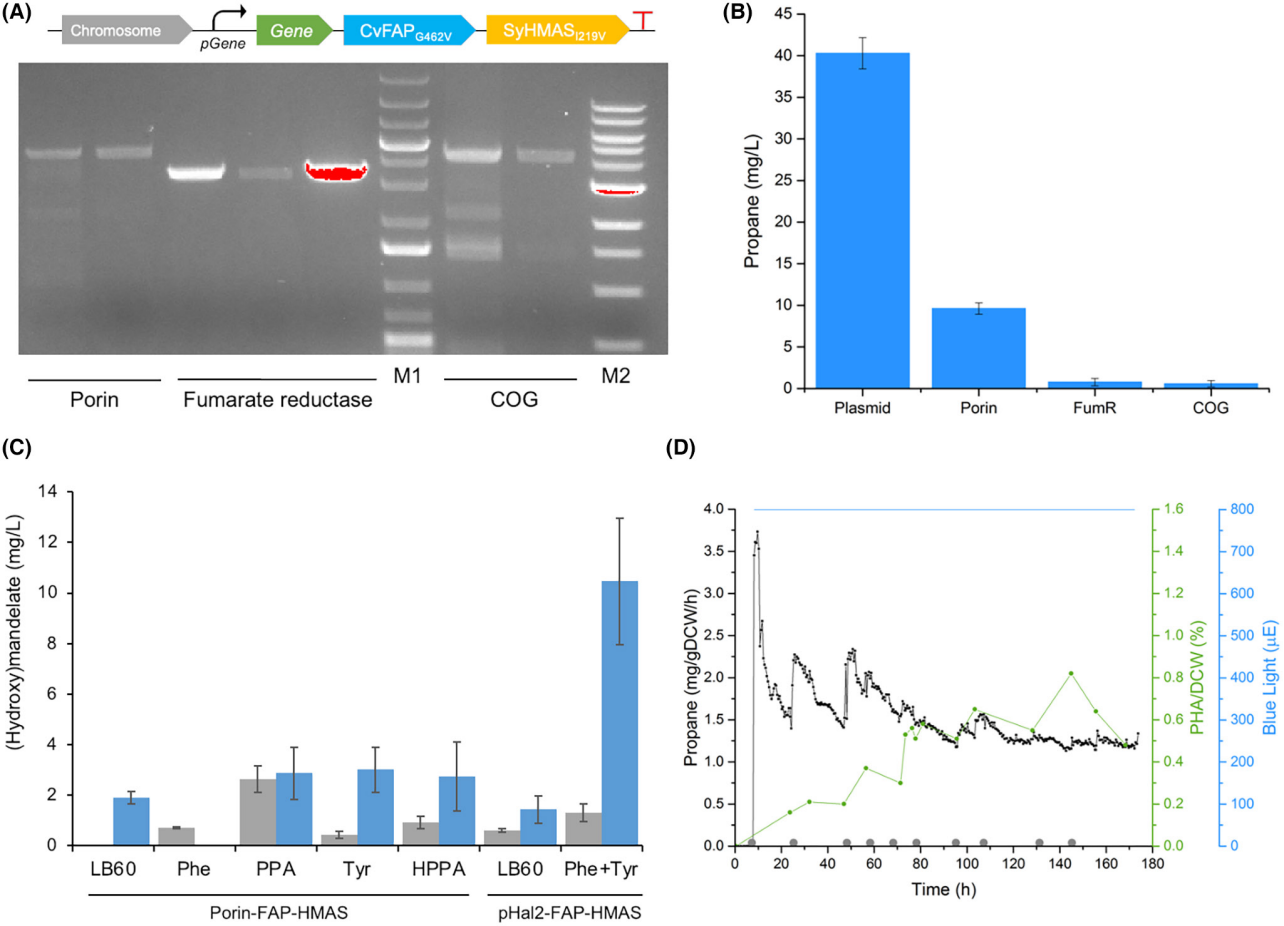
Genomic integration of CvFAP_G462V_ and SyHMAS_I219V_ at three sites in *Halomonas bluephagenesis* TD1.0. (A) Agarose gel electrophoresis of PCR reactions confirming the FAP‐HMAS_I219V_ insertion into the porin (porin), fumarate reductase (FumR) and universal stress protein COG0589 sites (COG). Positive band sizes are 4400 bp, 3300 bp, 4200 bp, respectively. Molecular mass marker M1 has band sizes of 12, 10, 8, 5, 4, 3, 2, 1.5, 1.25, 1 kbp. Marker M2 has band sizes of 10, 8, 6, 5, 4, 3, 2, 1.5, 1, 0.75 kbp. (B) Propane production of the genomic integrated constitutive FAP‐HMAS_I219V_ cassette at three loci compared to the plasmid‐borne IPTG‐inducible system. Cultures (1 ml) were grown in sealed vials overnight at 30°C under blue light. Propane headspace was measured using a Micro GC. Errors represent one standard deviation from triplicate samples. (C) Benchmark testing for (hydroxy)mandelate production in *H. bluephagenesis* TD1.0 containing genomic integrated por‐FAP‐HMAS or wild‐type containing plasmid pHal2‐T7‐FAP‐HMAS. Cultures (30 ml) were cultivated in LB60 medium containing 20 mM PPA, 20 mM HPPA, 3 g/L Phe, 3 g/L Tyr, or no supplementation, for 48 h at 30°C. (Hydroxy)mandelate concentration was determined by LCMS. (D) Fermentative propane and PHA production of *H. bluephagenesis* TD1.0 containing genomic integrated Por‐FAP‐HMAS_I219V_. The culture (400 ml) was grown in LB60 pH 6.8 at 30°C. Butyric acid (50 mM) was added 1 h after the OD 680 nm reached 0.8, with constant blue light illumination (800 μE; 8 h until end). Cultures were maintained with constant aeration (1.25 L/min) and temperature for 174 h. Butyric acid (~10 mmol) was added periodically, as indicated by the grey circles. Online headspace gas analysis for propane production was performed using a Micro GC, while PHA estimations were performed offline using a GC.

To generate an industrially relevant and genetically stable multi‐product microbial host it is preferable to integrate both SyHMAS_I219V_ and CvFAP_G462I_ directly onto the chromosome. We integrated these two genes into *H. bluephagenesis* TD1.0 by homologous recombination at three loci (Figure 5A). The chosen loci were downstream of the constitutively expressed porin (Por; Li, Li, et al., 2016), iron–sulphur fumarate reductase frdB (FumR; Cecchini et al., 2002) and the universal stress protein COG0589 (COG; Tkaczuk et al., 2013). Selection was based on the need to eliminate IPTG induction, with the strength of each promoter estimated from preliminary transcriptomics data of *H. bluephagenesis* TD1.0 from prolonged fermentations (Tsinghua University; unpublished results).

Propane and (hydroxy)mandelate production were measured for each *H. bluephagenesis* TD1.0 strain containing an integrated CvFAP_G462I_‐SyHMAS_I219V_ construct (Figure 5A). The highest propane titres were seen with the construct integrated at the strong porin promoter region (pPor‐FAP‐HMAS_I219V_; 9.61 ± 0.69 mg/L propane, <3 mg/L hydroxymandelate; Figure 5B,C). As expected, these titres are four‐fold lower than the strain expressing the equivalent plasmid‐borne construct (40.28 ± 1.89 mg/L propane), due to the decrease in copy number between a highly expressing plasmid and a single copy genomic insertion (40.28 ± 1.89 mg/L propane, 10.45 ± 2.50 mg/L hydroxymandelate). These reduced titres were obtained even in the presence of supplementation with (hydroxy)mandelate precursors phenylpyruvate, hydroxyphenyl pyruvate, phenylalanine or tyrosine.

We tested the longevity of productivity of the genome integrated *H. bluephagenesis* TD1.0 strain (pPor‐FAP‐HMAS_I219V_) by monitoring propane and PHA production during a 7‐day photobioreactor cultivation with periodic feeding of butyrate and constant blue light illumination (Figure S2). We found the same characteristic propane peak rate around 2.5 h after illumination commenced (3.7 mg/gDCW/h), but unlike plasmid‐borne systems only a slow decline in average propane production rates was seen (2.3–1.1 mg/gDCW/h; Figure 5D). Throughout the fermentation spikes in propane production corresponded to butyrate feeding, with significant propane production remaining after 7 days. Moreover, PHA accumulated steadily throughout the fermentation, reaching 82% g/g DCW (Figure 5D), suggesting butyrate was acting as an alternative carbon source.

A second 16‐day semi‐continuous fed‐batch cultivation was performed with intermittent cell harvest periods (95% cell harvest for PHA with fresh medium addition and dark cultivation). Propane production spikes were seen after each ‘dark’ cultivation, averaging out to 1.0 mg/gDCW/h, with a PHA content of 64 ± 3 g/g DCW (%; Figure S5). This highlights the stability of the genome integrated system and the potential for the development of an extended continuous fermentation system. The upper time limit may be in part determined by a blue light‐induced decrease in cell viability over time in the presence of butyric acid (Figure S6). Further discussion on blue light‐induced cell viability can be found in the Supplementary Results and Discussion section.

### Chassis engineering for increased (hydroxy)mandelate production

We have shown that the multiproduct engineered *H. bluephagenesis* TD1.0 strain generates high titres of PHA and the highest rate of propane production seen during continuous fermentations. However, (hydroxy)mandelate production is significantly lower than titres seen with optimized engineered *E. coli*. Therefore, in an attempt to boost (hydroxy)mandelate titres we incorporated a high copy number inducible plasmid containing the wild‐type or I219V variant of SyHMAS to the genome engineered strain (Figure 3). This strain showed high level expression of SyHMAS_I219V_ (and CvFAP; Figure S7A) and generated (*S*)‐hydroxymandelate with high enantiopurities (>99% ee; Figure S8). Mandelate titres (14.01 ± 0.64 mg/L) were improved compared to *H. bluephagenesis* TD1.0 containing only plasmid borne HMAS_I219V_ (Figure 3 inset), with similar hydroxymandelate titres (29.14 ± 3.12 mg/L).

We next sought to modify the genome of *H. bluephagenesis* to boost the intracellular accumulation of shikimate‐pathway precursors to further increase (hydroxy)mandelate titres. However, we were unable to identify an *E. coli*‐like phenylalanine and tyrosine degradation pathway targeted in previous studies (Robinson et al., 2020; Sun et al., 2011) nor a phenylalanine hydroxylase able to interconvert Phe and Tyr in the *H. bluephagenesis* genome. However, analogues of the *Pseudomonas putida* Phe and Tyr degradation pathway from homogentisate to fumarate and acetoacetate were identified (*hmgCAB*; Figure 1; Arias‐Barrau et al., 2004). We generated a knockout of the *hmgCAB* operon in *H. bluephagenesis* Por‐FAP‐HMAS_I219V_ (*hmgCAB* ^−^; Figure S7B,C), eliminating enzymes homogentisate 1,2‐dioxygenase (*hmgA*), 4‐maleylacetoacetate isomerase (*hmgB*) and fumarylacetoacetate hydrolase (*hmgC*). This strain was conjugated with the pHal2‐HMAS plasmid (wild‐type and I219V variant) and showed a 2.7‐fold improvement in mandelate titres between pHal2‐HMAS WT and I219V variants (10.86 ± 3.58 and 3.99 ± 0.05 mg/L, respectively). In contrast, hydroxymandelate titres were the same between the WT and I219V variant strain (39.65 ± 9.44 and 40.41 ± 1.96 mg/L respectively). This led to an overall titre increase and change in the ratio of hydroxymandelate to mandelate from 2.1 to 10.1 when the pHal2‐HMAS_I219V_ plasmid was present.

### (Hydroxy)mandelate production optimization using a DOE approach

Further optimization studies utilized a statistical design of experiment (DOE) approach to evaluate multiple parameters likely to affect titres of (hydroxy)mandelate production. Five key parameters identified from earlier scoping were varied in a 17‐culture definitive design screen (Table S3). Variables included growth temperature, optical density at induction and the concentrations of glucose (carbon source) and (hydroxy)mandelate precursors tyrosine and phenylalanine. Statistical modelling on experimentally observed hydroxymandelate titres suggested phenylalanine and tyrosine concentrations may significantly affect final titres (*p*‐values of 0.0122 and 0.0191 respectively; Figure S8). A second more focused screen with increased replicates was performed varying only the concentrations of glucose, tyrosine and phenylalanine (Table S4). A more precise statistical model was generated (Figure S9), predicting initial tyrosine (*p*‐value = 0.00002) and to a lesser degree glucose concentrations (*p*‐value = 0.0224) were likely to influence hydroxymandelate titres. The predicted ‘optimal’ supplemental concentrations were 4.7 and 11.6 g/L, respectively, although tyrosine would need to be supplemented throughout the fermentation to alleviate its solubility issues.

### Fermentative co‐production of four products by *H. bluephagenesis* TD1.0

We sought to optimize the fermentation conditions for multi‐product generation by *H. bluephagenesis* TD1.0 Por‐FAP‐HMAS_I219V_ *hmgCAB* ^−^ strain with pHal2‐HMAS_I219V_ beyond proof‐of‐principle demonstration. Multiple fermentations were performed to scope feeding regime, run length and harvesting strategies along with eight other physical process parameters. Five representative runs are reported here (Table 1) and full details of the methodology, monitoring and analytics of each bioprocess run are detailed in Figures S10–S14. Process conditions held constant were culture temperature (30°C; not statistically significant by DOE) and pH maintenance between 6.6 and 7, as required for CvFAP activity.

**TABLE 1:** Multi‐product fermentation of *Halomonas bluephagenesis* por‐FAP‐HMAS_I219V_ *HmgCAB* ^−^ with pHal2‐HMAS_WT or I219V_

Condition	Run 1	Run 2	Run 3	Run 4	Run 5
*Fermentation variables*					
Plasmid HMAS variant	WT	I219V	I219V	I219V	WT
Induction OD 600 nm	4.6	5.4	4.9	11.0	13.3
Light (μE)	800	800	800	800	400
Aeration (L/min)	1.25	0.3	0.48	1.25	1.25
Glucose (g/L)	30	0	3	10	0
Butyrate (g/L)	60	120	60	60	30
Tyr (g/L)	0	1.5	0.5	0.5	0.5
Phe (g/L)	1.5	0	1.5	0.5	0.5
Final culture OD 600 nm	16	8	5.6	17	17.4
Run length (h)	65	94	79	100	80
*Product titres*					
Harvest PHA (g/gDCW %)	70.5 (82.4)	32.5 (32.8)	63 (26.6)	77.1 (67.7)	34.9 (41.4)
Cumulative propane (mg/gDCW)	29.4	69.0	116.3	74.1	65.2
Hydroxymandelate (mg/gDCW)	2.74 ± 0.35	8.34 ± 0.09	2.88 ± 0.96	7.93 ± 0.74	35.32 ± 4.95
Mandelate (mg/gDCW)	21.97 ± 0.69	1.92 ± 0.06	12.03 ± 0.81	19.53 ± 0.28	87.34 ± 2.34

*Note*: Each culture (400 ml) was grown in LB60 pH 6.8 at 30°C in a photobioreactor with constant optical density monitoring (OD 680 nm), pH control, agitation and aeration. Offline analytics for metabolite monitoring were performed using HPLC and GC. Full details of the fermentation medium, running conditions, cell viability, and monitoring/analytical data for each run can be found in Figures S10–S14. PHA data in parentheses = after 20 g/L glucose addition followed by a 3‐h incubation.

Overall, we found a positive correlation between total glucose addition and PHA concentration (Table 1), particularly when glucose concentrations were high during harvesting (run 1: 70.5 g/gDCW (%) PHA; Figure S10). The lowest PHA titres (32.5 g/gDCW (%)) were seen when no glucose was added to the medium. These results are not surprising given that glucose (or excess simple carbon source presence) is a known stimulus for PHA production (Jendrossek & Pfeiffer, 2014).

Increased titres of mandelate were correlated with the supplementation of Phe, with a less noticeable effect (and final titre) seen between hydroxymandelate and Tyr (Table 1). Increasing the aeration rate appeared to correlate with higher mandelate, but not hydroxymandelate titres. Induction at a higher OD 600 nm increased the production of mandelate and hydroxymandelate combined, especially when the light intensity was halved. There was also an apparent negative correlation between (hydroxy)mandelate production and butyrate concentration (Table 1).

Cumulative propane production showed an apparent correlation with glucose concentration (Table 1). Propane levels increased with glucose concentrations between 0 and 3 g/L (max 116.3 mg/gDCW), then systematically decreased with excess glucose (up to 30 g/L) down to 29.5 mg/gDCW. Other process conditions did not seem to impact propane titres significantly, likely due to its rapid removal from the culture via the fermentation exhaust gas shortly after its formation.

**FIGURE 6:**
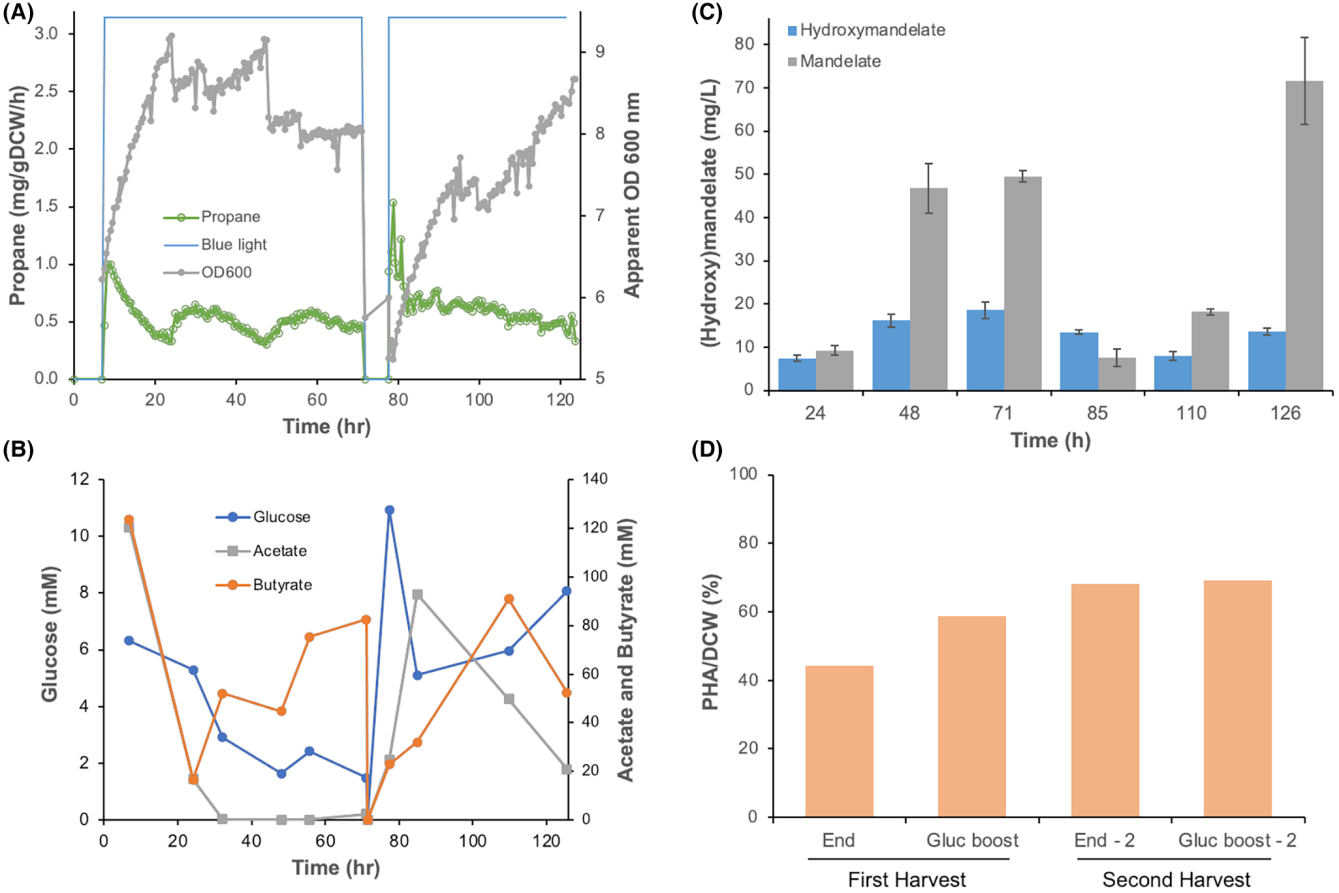
Fermentative production of propane, PHA, mandelate, and hydroxymandelate by *H. bluephagenesis* Por‐FAP‐HMAS_I219V_ *HmgCAB* ^−^ with pHal2‐HMAS_I219V_. The culture (400 ml) was grown in LB60 pH 6.8 at 30°C with 1.25 L/min aeration. The fermentation consisted of two batches with a dark harvest period at 70 hours and 126 hours. In the first and second batch after OD 600 nm reached 9 and 5.6 respectively, butyric acid (25 mM) was added and the blue light (400 μE) was switched on 1 h later. During harvest ~350 culture was removed for PHA harvesting, with % PHA shown as orange diamonds. At this time fresh LB60, antibiotic, glucose, tyrosine and phenylalanine were added to allow the culture to grow. Between harvests the culture was maintained with butyric acid (25 mM) twice daily. Offline analytics for metabolite monitoring were performed using HPLC and GC. (A) Propane and culture optical density are shown as green and grey circles, respectively. The timing of the blue light is shown schematically as a blue line, with dark phases shown as gaps. (B) HPLC analysis of glucose, acetate and butyrate throughout the fermentation. (C) Mandelate (magenta squares) and hydroxymandelate (blue squares) titres and (D) PHA production during fermentation. Apparent OD 600 nm = photobioreactor optical density probe data corrected for non‐linearity using the calibration curve in Figure S16.

In summary, optimization parameters for elevated multi‐product titre were identified as: (i) induction at a high optical density, (ii) high oxygenation, (iii) reduction in light exposure, (iv) reduction in butyric acid feeding, (v) regular feeding of Phe or Tyr dependent on the desired product, (vi) reduction in glucose feeding during the main fermentation followed by a spike prior to harvesting, and (vii) utilizing a semi‐continuous fed batch approach. We combined these parameters and performed a sixth fermentation to make all four compounds (Figure 6; Figure S15). This included two consecutive batch runs with 95% culture harvesting followed by fresh medium addition and a 6‐h dark harvest period for biomass accumulation in the absence of propane production. At the start of each batch, we supplemented both Phe and Tyr for (hydroxy)mandelate, glucose for PHA and culture growth and butyric acid for propane production. An additional glucose feed was performed close to each harvest to maximize PHA accumulation.

Polyhydroxyalkanoates titres of 69 g/gDCW (%) were achieved after the second culture harvest, similar to batch culture studies. Propane production showed the characteristic early spike after illumination, followed by a typical slow decline (1.53 mg/gDCW/h dropping to 0.33 mg/gDCW/h after 5 days; Figure 6). Mandelate production dominated over hydroxymandelate and showed titres close to the maximal achieved by *H. bluephagenesis* to date (71.63 + 10.03 mg/L). Therefore, while small differences in some product titres are seen in this combined fermentation process, overall, we saw a general maintenance of productivity compared to each compound titre generated in isolation.

### Downstream processing of (hydroxy)mandelate

Key to our proof‐of‐concept cost‐effective multi‐product process is the ease of separation of each product post fermentation to minimize downstream processing costs. Propane (gas) and PHA (insoluble cellular component) are easily separated using industry standard procedures, however, both mandelate and hydroxymandelate are secreted into the culture supernatant. Therefore, to separate soluble mandelate and hydroxymandelate from the supernatant, we tested a simple and cost‐effective selective recrystallization method from water (Xiao et al., 2020). Using concentrations similar to those found during fermentation, we achieved a purity of mandelate to hydroxymandelate from 85% to 94% after only one round of recrystallization. Therefore, simple and low‐cost techniques are available to purify the desired compound post fermentation, with iterative rounds of recrystallization likely to improve the purity further.

## DISCUSSION

We hoped to minimize co‐production complexity through picking chemical targets that could be easily separated, that had minimal competition for pathway intermediates, and that required minimal genetic modification to maintain host health and growth rates. These requirements were met by selecting native PHA production, recruiting the Shikimate pathway for (hydroxy)mandelate production (just one recombinant enzyme step) and the addition of one‐step bio‐propane production with a waste organic carbon feed. We found PHA and propane co‐production was convenient and reliable, but that optimization of all four products could lead to situations where parameters increasing the production of one compound reduced another. Therefore, the main challenge of this study was to establish (hydroxy)mandelate production in *H. bluephagenesis* TD1.0 and optimize the fermentation system to minimize any impact co‐production of multiple products had on each individual titre.

### PHA production

We found high titres of PHA (up to 72 g/gDCW %) occurred when cells were harvested under high and steady glucose conditions. This is consistent with the knowledge that PHA production is generally stimulated by the presence of high levels of carbon source (high carbon to nitrogen ratio), with rapid depolymerization when available carbon sources were depleted (Figure 2). In addition, we found that butyrate supplementation also influenced PHA production, presumably by acting as a secondary carbon source.

A glucose feed in carbon depleted cultures 2 h prior to harvesting was found to be ineffective at significantly increasing PHA accumulation (Figures S10–S15) suggesting maintenance of a significant level of glucose throughout the fermentation is more critical. Butyrate depletion was seen at rates higher than propane production, appearing to correlate with PHA accumulation. This suggests butyrate acts as a secondary carbon source for *H. bluephagenesis* TD1.0 and must be maintained at high concentrations to ensure enough is available to act as propane precursor. Scoping of butyrate as the single precursor for both compounds warrants further investigation as an inexpensive alternative carbon source to glucose.

### Propane production

This study demonstrates the highest levels of bio‐propane production to date within a *Halomonas* species. It was encouraging to find that the wild‐type TD1.0 produces higher propane titre and cell density than a PHA‐deficient strain, suggesting PHA production leads to healthier, more metabolically active cells. PHA may also act as a protective barrier from UV radiation, maintaining cell viability and ability to replenish the photobiocatalyst throughout an extended fermentation. As propane levels never accumulate within the photobioreactor (expelled in the exhaust gas) any impact of fermentation conditions likely impacts the functional expression and maintenance of activity of CvFAP_G462I_. As exposure of blue light is generally cytotoxic, a more suitable approach to prolonged fermentations may be to alternate between a constant illuminated fed‐batch cultivation and periodic harvesting, feeding and ‘dark’ fermentation cycles to replenish the live cells (and active CvFAP_G462I_). This periodic harvest, replenish and light cycles should reduce the costs associated with an alternative full harvest and reload, provided the time spent replenishing the biocatalyst does not significantly reduce the overall yields of propane.

Genome integration of CvFAP_G462I_ led to a prolonged high propane production over 7 days and allowed extended (28 day) productive fermentations to be possible in the absence of antibiotics. Further studies are required in more customized scaled photobioreactors to determine if this process is scalable, to ensure sufficient light penetration is possible to balance photocatalyst activation (propane production) with the detrimental effects on cell viability and culture growth.

### (Hydroxy)mandelate production

This is the first published example of (*S*)‐(hydroxy)mandelate production in *Halomonas*. We found that purified wild‐type SyHMAS and I219V variant generated (*S*)‐hydroxymandelate with high enantiopurity, but with overall titres reduced compared to in vivo *E. coli* studies using these enzymatic variants. We suspect a factor limiting (hydroxy)mandelate production is a low flux through the shikimate pathway and utilization of Tyr/Phe as carbon sources in *Halomonas*. We produced genetic knockouts to increase intracellular pathway precursors Phe and Tyr by generating a *hmgCAB* ^−^ knockout strain. We also incorporated stable genome integration with an additional enzyme ‘boost’ plasmid and performed multiple DOE(s) to adjust fermentation process conditions. Despite these efforts, we were unable to achieve *E. coli*‐like titres of (hydroxy)mandelate, but have provided a proof‐of‐principle demonstration of production in *Halomonas* by incorporating only one recombinant enzyme. Therefore, further improvements are needed to evolve this ‘proof of principle’ demonstration to achieving titres exceeding those observed by multi‐knockout recombinant *E. coli* system.

## CONCLUSIONS

Multi‐product fermentation has the potential to maximize profits and minimize costs, by utilizing all nutrients in a feedstock, obviating energy and power spent on multiple reactors, and producing multiple sellable value‐added products. We have achieved the production of four products: a gaseous biofuel, two soluble extracellular biochemicals, and a cell‐bound bioplastic PHB. Due to the inherent phase uniqueness of most of the products, their separation is simplified. Propane is collected from the headspace and can be liquified for transport or used directly as fuel using existing infrastructures. PHA is harvested from the cell mass after centrifugation from the broth. Finally, (hydroxy)mandelate is extracted from the liquid phase, with re‐crystallization as a viable purification technique for separation of the two soluble products.

This study has highlighted *H. bluephagenesis* TD1.0 as a suitable chassis to produce high levels of PHA and demonstrated the highest reported titres of bio‐propane. Realization of the commercial viability of this multiproduct bioproduction processes will necessitate an increase in titre of (hydroxy)mandelate, or the substitution with another high‐value water‐soluble product of high titre. Strategies for increased productivity could be achieved by optimization of scaled fermentation conditions. Chassis redesign is also key to improving recombinant enzyme expression and knockdown of competing pathways.

Additional considerations are needed for scaled co‐production strategies utilizing photocatalysts, including balancing light stimulation of chemical production with catalyst poisoning, cell growth and viability. We envision a scaled process that would require a multi‐stage approach, involving initial biomass production in a standard low‐cost medium in the absence of light. This would be followed by the addition of propane and (hydroxy)mandelate precursors and blue light illumination until cell viability and/or biocatalyst activity is significantly affected. A steady glucose feed would be supplied to initiate and maintain a high PHA concentration throughout the fermentation. Periodic harvests and feeding and cultivation in the absence of light would replenish biocatalyst‐containing cells depleted by illumination without the need of a costly full harvest and reload.

Overall, this study has shown that the co‐production of multiple valuable biomolecules is possible within one fermentation. The selection of easily separatable high‐value products of high titre is key to tipping the balance of microbial bioproduction strategies towards commercial viability. This approach could boost further study and investment into the extensive library of proof‐of‐principle, yet low titre bioproduction routes, supporting the evolving bioeconomy with cost‐competitive sustainable and renewable routes towards chemicals and fuels.

## AUTHOR CONTRIBUTIONS

Nigel S. Scrutton, Guo‐Qiang Chen, Helen Park and Helen S. Toogood conceived of initial objective to create multiple products in a fermentation. Nigel S. Scrutton, Guo‐Qiang Chen and Helen Park selected target compounds. Helen Park performed experimental work. Helen Park and Helen S. Toogood analysed and compiled experimental results, and drafted the manuscript.

## CONFLICT OF INTEREST

Nigel S. Scrutton and Helen S. Toogood have affiliations with C3 Biotechnologies Ltd, which has commercial interests in production of gaseous hydrocarbon fuels.

## Supporting information


Appendix S1

## Data Availability

All experimental data pertinent to a review of the manuscript are contained within the manuscript.
