# Supplementary material for: Co‐production of biofuel, bioplastics and biochemicals during extended fermentation of Halomonas bluephagenesis
Source: Microb Biotechnol. 2022 Nov 9;16(2):307–21. doi: 10.1111/1751-7915.14158 (PMC9871518; doi:10.1111/1751-7915.14158)
Supplement: Supplementary file 1 — Appendix S1 [file MBT2-16-307-s001.pdf]

Supplementary Information

Co-production of biofuel, bioplastics, and biochemicals during extended fermentation of *Halomonas bluephagenesis*

Helen Park,[a,b] Helen S. Toogood,[a] Guo-Qiang Chen[b] and Nigel S. Scrutton[a*]

[a]EPSRC/BBSRC Future Biomanufacturing Research Hub, EPSRC Synthetic Biology Research Centre SYNBIOCHEM Manchester Institute of Biotechnology and School of Chemistry, The University of Manchester, Manchester, M1 7DN, UK.

[b]Center for Synthetic and Systems Biology, School of Life Sciences, Tsinghua-Peking Center for Life Sciences, Tsinghua University, Beijing 100084, China

[*]Corresponding author: nigel.scrutton@manchester.ac.uk

Contents

Supplementary Experimental 3

Supplementary Results and Discussion 5

Supplementary Figures

Fig. S1 Schematic view of the A) plasmid-borne and B) genomic-integrated constructs to produce propane and/or (hydroxy)-mandelate. 7

Fig. S2 Enantiopurity determination of hydroxymandelate and mandelate by LCMS. 8

Fig. S3 Growth of *H. bluephagenesis* TD1.0 on medium with glucose or butyric acid as a sole carbon source. 9

Fig. S4 Fermentative propane production by *H. bluephagenesis* TD1.0 containing genomic integrated Por-FAP-HMAS$_{I219V}$ at two pH values. 10

Fig. S5 Fermentative propane and PHA production of *H. bluephagenesis* TD1.0 containing genomic integrated Por-FAP-HMAS$_{I219V}$. 11

Fig. S6 Effect of blue light and butyric acid on the A) growth and B) cell viability of *H. bluephagenesis* TD1.0 Por-FAP-HMAS$_{I219V}$. 12

Fig. S7 Qualitative analysis of CvFAP and HMAS expression and *hmgCAB* knockout in *H. bluephagenesis* TD1.0. 13

Fig. S8 Summary of the first set of Design of Experiment (DOE) calculations for hydroxymandelate production. 14

Fig. S9	Summary of the second set of Design of Experiment (DOE) calculations for hydroxymandelate production.	15
Fig. S10	Fermentative production of propane, (hydroxy)mandelate and PHA by *H. bluephagenesis* Por-FAP-HMAS$_{I219V}$ *HmgCAB*$^{-}$ with pHal2-HMAS$_{WT}$ (Run 1).	16
Fig. S11	Fermentative production of propane, (hydroxy)mandelate and PHA by *H. bluephagenesis* Por-FAP-HMAS$_{I219V}$ *HmgCAB*$^{-}$ with pHal2-HMAS$_{I219V}$ (Run 2).	17
Fig. S12	Fermentative production of propane, (hydroxy)mandelate and PHA by *H. bluephagenesis* Por-FAP-HMAS$_{I219V}$ *HmgCAB*$^{-}$ with pHal2-HMAS$_{I219V}$ (Run 3).	18
Fig. S13	Fermentative production of propane, (hydroxy)mandelate and PHA by *H. bluephagenesis* Por-FAP-HMAS$_{I219V}$ *HmgCAB*$^{-}$ with pHal2-HMAS$_{I219V}$ (Run 4).	19
Fig S14	Fermentative production of propane, (hydroxy)mandelate and PHA by *H. bluephagenesis* Por-FAP-HMAS$_{I219V}$ *HmgCAB*$^{-}$ with pHal2-HMAS$_{I219V}$ (Run 5).	20
Fig. S15	Fermentative production of propane, (hydroxy)mandelate and PHA by *H. bluephagenesis* Por-FAP-HMAS$_{I219V}$ *HmgCAB*$^{-}$ with pHal2-HMAS$_{I219V}$ (Final optimized run).	21
Fig. S16	Calibration curves for the conversion of A) photobioreactor optical density probe (680 nm) to spectrophotometer OD 600 nm and B) OD 600 nm to dry cell weight of *H. bluephagenesis*.	22

Supplementary Tables

Table S1	Expression constructs for *H. bluephagenesis* TD1.0 and genome integrated variants in this study.	23
Table S2.	Oligonucleotide sequences for the assembly of plasmids used in this study.	24
Table S3.	DOE parameters and hydroxymandelate production for the first screen with *H. bluephagenesis*.	25
Table S4.	DOE parameters and hydroxymandelate production for the second screen with *H. bluephagenesis*.	26
**References**		26

Supplementary Experimental

Materials, strains, plasmids and growth medium

Media components were purchased from Formedium (Norfolk, UK). The *E. coli* strains used for plasmid construction and propagation were Stellar (Clontech) and NEB5α (New England Biolabs). The standard growth medium for *E. coli* was Luria broth (LB; 5 g/L yeast extract, 10 g/L tryptone and 10 g/L NaCl pH 7.0). The concentration of spectinomycin, kanamycin, chloramphenicol and tetracycline used in antibiotic-selection media was 50, 30, 34 and 10 μg/mL, respectively. Gene sequencing and oligonucleotide synthesis were performed by Eurofins MWG (Ebersberg, Germany).

The pSH-based plasmids contain the CRISPR/Cas9 editing *H. bluephagenesis* genome donor DNA pSEVA241 plasmid (Qin *et al.*, 2018), with the gRNA and antibiotic resistances removed. The latter plasmid contains oriT, I-SceI meganuclease gene under *trc* control and a pRO1600-ColE1 double origin (Amer *et al.*, 2020b). The backbone is based on pSEVA and Biobrick vectors and is required to facilitate genomic DNA integration.

Genomic integration and hmgCAB knockout

All genomic insertion and deletions were performed via homologous recombination using a previously published suicide vector protocol (Amer *et al.*, 2020b). In this methodology, the pSH-based Por/FumR/COG-FAP-HMAS$_{I219V}$ constructs or the knockout pHal2-tet-hmgH1H2 plasmid are co-expressed within *H. bluephagenesis* TD1.0 with a second kanamycin and spectinomycin-resistant plasmid pSBR1Ks-i-SceI. Plasmid conjugation into *H. bluephagenesis* TD1.0 was performed sequentially (pSBR1Ks-i-SceI first) using the modified protocol described previously (Amer *et al.*, 2020a, Amer *et al.*, 2020b). The pSH-based plasmids were conjugated last as they contain a ColE1 *ori* that is not replicated in *H. bluephagenesis*. *In vivo* expression of I-SceI by the addition of IPTG (0.1 M) induces the linearization of pSH plasmids. Once linearized, this suicide vector will integrate FAP-HMAS$_{I219V}$ or the Tet^R cassette into the genome at the location complementary to the plasmid homology arms (Fu *et al.*, 2014, Qin *et al.*, 2018). Successful integration of the FAP-HMAS$_{I219V}$ cassette was seen as growth of *H. bluephagenesis* on chloramphenicol-selective LB60 medium (tetracycline for successful *hmgCAB*$^-$ knockout), as the pSH plasmid is not replicated in *H. bluephagenesis*. Integration was confirmed by colony PCR, genomic sequencing, and *in vivo* propane production after pSceI plasmid curing (Fu *et al.*, 2014, Qin *et al.*, 2018).

Optimization of secondary product generation using design of experiment (DOE)

Optimization of (hydroxy)mandelate production was performed using a statistical design of experiment (DOE) approach to evaluate multiple parameters likely to affect titers with reduced experimental effort. The key parameters identified from experimental observations during multiproduct fermentations were: *i*) temperature; *ii*) optical density at induction and the *iii*) concentrations of glucose (carbon source) and the (hydroxy)mandelate precursors tyrosine and phenylalanine. The software JMP® Pro Statistical Software (SAS institute, North Carolina, USA) was used to create a definitive experimental design screen. In this screen, all five parameters were varied, and 17 individual growth conditions were identified for comparative

in vivo testing (**Supplementary Table S4**). A second more focused screen was performed, based on the initial screen, that varied only the concentrations of glucose, tyrosine and phenylalanine (**Supplementary Table S5**).

Testing of the individual DOE conditions was performed for *H. bluephagenesis* Por-FAP-HMAS$_{I219V}$ *HmgCAB*$^-$ strain with pHal2-HMAS$_{WT}$ in LB60 pH 6.8 containing 50 µg/mL spectinomycin. The initial culture (600 mL) was incubated at 30°C with 180 rpm agitation until the required OD 600 nm was achieved (1, 5.5 or 10; **Supplementary Table S4-S5**). Culture aliquots (30 mL) were dispensed into individual flasks and the required amounts of glucose, tyrosine and phenylalanine were added together with 0.1 M IPTG. Cultures were incubated for 48 h at the designed temperature (22, 30 or 37°C; **Supplementary Table S4-S5**), followed by (hydroxy)mandelate analysis by LCMS as above. Statistical modelling was performed on the (hydroxy)mandelate titer data by least squares linear regression (LSLR), a method that finds the best fit for the data by minimizing the sum of residuals, generating a line of best fit that can predict (hydroxy)mandelate titers (Gilman *et al.*, 2021). The experimental vs. model prediction plot, residual plot and other calculations for each study can be found in **Supplementary Figures S4-S5**. The p-value was used to judge significance of each parameter, by testing the null hypothesis that the coefficient for each factor is zero. A p-value < 0.05 indicates we can reject the null hypothesis and that the factor is significant. The LSLR model could then be used as a directional tool to suggest optimal factor values in subsequent fermentations and limited the scope of factors to test.

Analytical techniques

Propane titers from small, sealed cultures were determined by manual headspace injection into an Agilent 490 Micro GC, containing an Al_2O_3/KCl column and a thermal conductivity detector (TCD) (Amer *et al.*, 2020b). For automated fermenter off gas propane detection, a continuous stream of headspace gas was passed through an ice-cooled condenser to remove the majority of water vapor prior to entry into the Micro GC sample cell. Analysis of the gas content was performed every 20 minutes, using running conditions described previously (Amer *et al.*, 2020b). Peak areas were compared to those from a gas standard composed of 1% each of propane, butane, and isobutane with a nitrogen balance (Thames Restek). Error bars indicate one standard deviation of the data obtained for the replicates (biological and/or technical triplicates).

PHA hydrolysis and methanolysis was performed by a modification of the method described previously (Ye *et al.*, 2018a). Dried cell pellet (20-40 mg) was dissolved in 1 mL chloroform within 4 mL airtight glass vials. The derivatization reagent (1 mL; 3% H_2SO_4 in anhydrous methanol containing the internal standard 0.1% benzoic acid) was added and incubated at 100°C in a sand bath for 4-6 h. After reaction completion, samples were cooled to room temperature, and ~1 g of sodium bicarbonate was added to quench the reaction. Tubes were re-sealed and placed in the fridge overnight to allow any cell debris to precipitate. Cells were spun down, and the liquid fraction was clarified by passage through a filter tip. Samples were analyzed for methyl 3-hydroxybutyrate content (derivatized PHA monomer) by GC using an Agilent Technologies 7890A GC equipped with an FID detector. Product(s) (1 µL) were separated on a HP-5 column (30 m x 0.32 mm i.d., 0.25 µM film thickness, Agilent

Technologies). In this method, the injector temperature was set at 250°C with a split ratio of 10:1. The carrier gas was helium with a flow rate of 10 mL/min and a pressure of 3.3 psi. The program began at 80°C (2 min hold), then ramped to 320°C at 20°C/min, with a final hold of 5 mins. Compound identification was determined by comparison of the retention time of authenticated standards. Quantitative analysis was performed using experimentally determined relative response factors in relation to the internal standard (0.1% benzoic acid). PHA content is expressed as the percentage of PHA mass as a fraction of the total dry cell weight (DCW) of the lyophilized cell pellet (% g/g DCW).

Aqueous culture metabolites (butyrate, acetate, glucose and glycerol) were analyzed by HPLC using an Agilent 1260 Infinity HPLC with a 1260 ALS autosampler, TCC SL column heater, a 1260 refractive index detector (RID). Samples were run on an Agilent Hi-Plex H column (300 x 7.7 mm; 5 mM H_2SO_4) using the running conditions described previously (Amer *et al.*, 2020b). Each analyte concentration was calculated by comparing the peak areas to a standard curve generated from analytical standards of known concentration. Error bars indicate one standard deviation of the data obtained for the replicates (biological and/or technical triplicates).

The relative cell viability of culture samples was determined using the CellTitre-Glo® 2.0 kit (Promega). A modified protocol was employed where the culture was normalized to an OD 600 nm of 1. The samples were incubated with 20 μL of CellTitre-Glo Reagent for 10 min at 37 °C with 800 rpm agitation. The luminosity of quadruple aliquots (20 μL) of each sample within 384-well white plates was measured using a BMG Labtech Clariostar plate reader. The program measured luminosity endpoint without a filter, using an emission spectrum range of 490-700.

Supplementary Results and Discussion

Effect of pH on propane production

Industrial-scale *H. bluephagenesis* TD1.0 cultivations are typically performed at high pH (Ye *et al.*, 2018b). However, prior studies showed that propane production would not occur in *H. bluephagenesis* TQ10 (a derivative of TD1.0) expressing CvFAP unless the pH was near neutral (Amer *et al.*, 2020a, Amer *et al.*, 2020b). Therefore, we tested the ability of *H. bluephagenesis* TD1.0 to generate propane at pH 6.8 and 9. This required butyrate feeds during all subsequent fermentations to be pH adjusted to the pH of the culture, rather than adding butyric acid direct into the culture. The results showed that propane production was not detectable at high pH (**Supplementary Figure S4**), so further work was performed in cultures maintained at pH 6.8, the optimum found for the TQ10 strain.

Effect of blue light on H. bluephagenesis cell viability

Earlier studies with *H. bluephagenesis* TQ10 showed a significant decrease in cell viability (detected via monitoring colony forming units throughout prolonged incubations) with constant blue light illumination within a photobioreactor (Amer *et al.*, 2020b). This limits the maximum fermentation time achievable without using a fed batch approach or performing

regular 'dark' cultivation cycles that allow surviving cells to replicate and regenerate the CvFAP catalyst. However, the TQ10 strain is a lower fitness strain compared to TD1.0, as the former is lacking the genes necessary to produce the carbon storage molecule PHA. Therefore, we investigated the effect of prolonged blue light exposure on *H. bluephagenesis* TD1.0 cell viability in the presence and absence of butyrate at pH 6.8.

In the absence of butyrate, no significant decrease in cell viability was seen whether blue light was present or not (**Supplementary Figure S5**). This contrasts to cells cultivated with supplemental butyric acid, which saw a significant decrease in cell viability when illuminated with blue light. This differs from the TQ10 strain, which showed an overall increase in loss of cell viability during prolonged light exposures. This apparent improved fitness of the TD1.0 strain may be due to the accumulation of PHA granules intracellularly, as this is known to scatter UV radiation and thereby provide a substantial UV-protection. This was seen with UV-challenged PHA-accumulating *Cupriavidus necator*, which also saw a decrease in the intracellular level of reactive oxygen species (Slaninova *et al.*, 2018).

Supplementary Figures

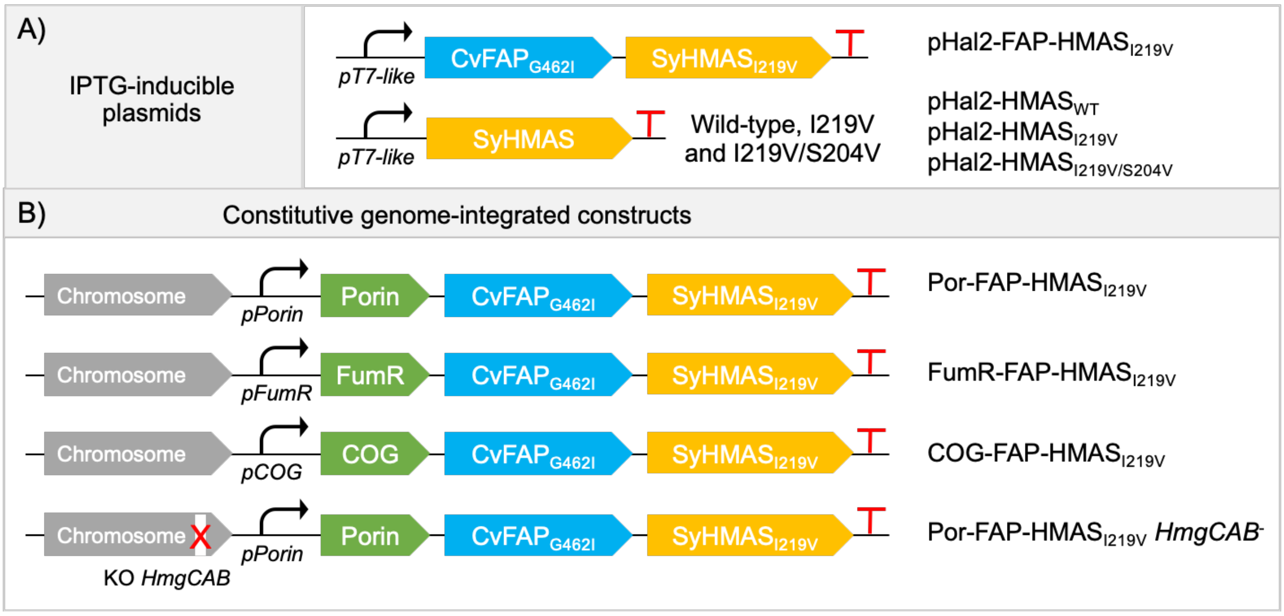

Figure S1. Schematic view of the A) plasmid-borne and B) genomic-integrated constructs to produce propane and/or (hydroxy)mandelate. Recombinant enzymes: CvFAP$_{G462V}$ or FAP = G462I variant of fatty acid photodecarboxylase from *Chlorella variabilis*; SyHMAS (WT and variants) = 4-hydroxymandelate synthase from *Streptomyces yokosukanensis*. *H. bluephagenesis* TD1.0 endogenous genes: Por = porin; FumR = fumarate reductase; COG = universal stress protein COG0589.

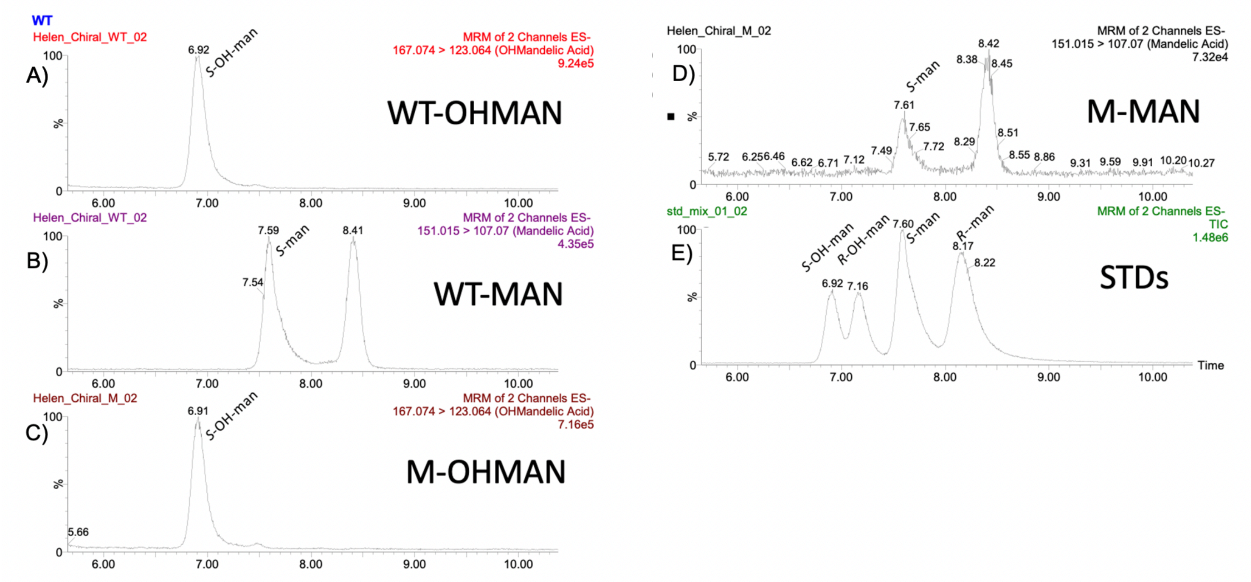

Figure S2. Enantiopurity determination of hydroxymandelate and mandelate by LCMS. Samples of mandelate and hydroxymandelate generated by *H. bluephagenesis* TD01 cultures containing (A-B) pHal2-HMAS$_{WT}$ and (C-D) pHal2-HMAS$_{I219V}$ plasmids. Cultures (50 mL) were cultivated in LB60 pH 6.8 containing containing 50 µg/mL spectinomycin for 12 h, followed by 0.1M IPTG induction. The cultures were incubated a further 48 h and 1 mL of each culture supernatant was removed for chiral HPLC analysis, as described previously (Robinson *et al.*, 2020).

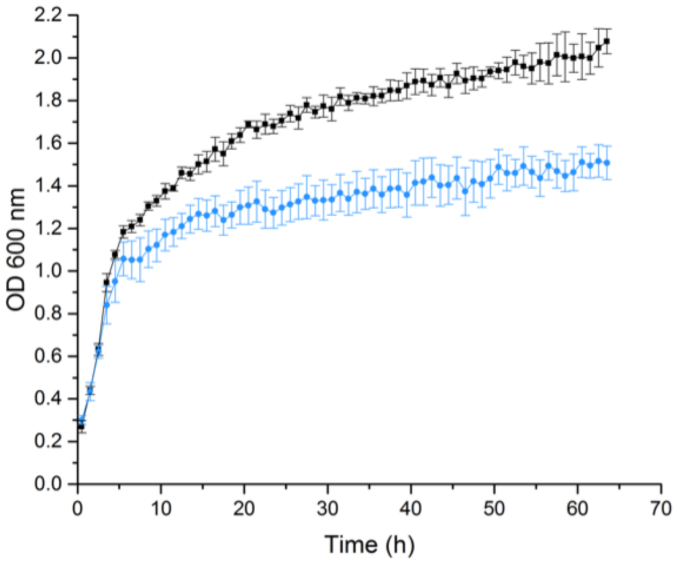

Figure S3. Growth of *H. bluephagenesis* TD1.0 on medium with glucose or butyric acid as a sole carbon source. Cultures were grown overnight and used to inoculate a 96-well microplate. Cultures (1 mL) were grown in high salt MM63 medium pH 9 with either 1 g/L glucose or 1 g/L butyric acid at 30 °C with shaking within a 96-well microplate. The OD 600 nm was monitored every 5 min for 64 h. The error bars represent one standard error from 6 replicates, and the 30-min interval readings are shown on the graph. Glucose and butyric acid data are shown as black and blue spheres, respectively.

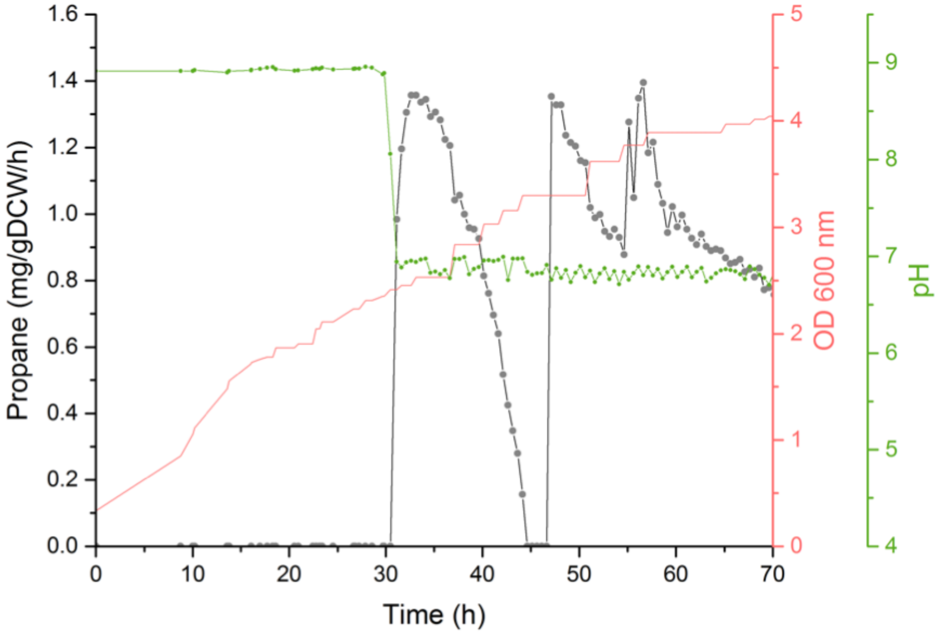

Figure S4. Fermentative propane production by *H. bluephagenesis* TD1.0 containing genomic integrated Por-FAP-HMAS$_{I219V}$ at two pH values. The culture (400 mL) was grown in LB60 pH 9 at 30 °C. Butyric acid (50 mM) was added 1 h after induction (9 h), with constant blue light illumination (800 µE, 10 h). Cultures were maintained with constant aeration (1.25 L/min) and temperature for ~ 30 h, then the pH was adjusted and maintained at 6.8 until 70 h had elapsed. Apparent OD 600 nm = photobioreactor optical density probe data corrected for non-linearity using the calibration curve in **Supplementary Figure S16**. Online headspace gas analysis for propane production was performed using a Micro GC. Discussion of this data is found in the **Supplementary Results and Discussion**.

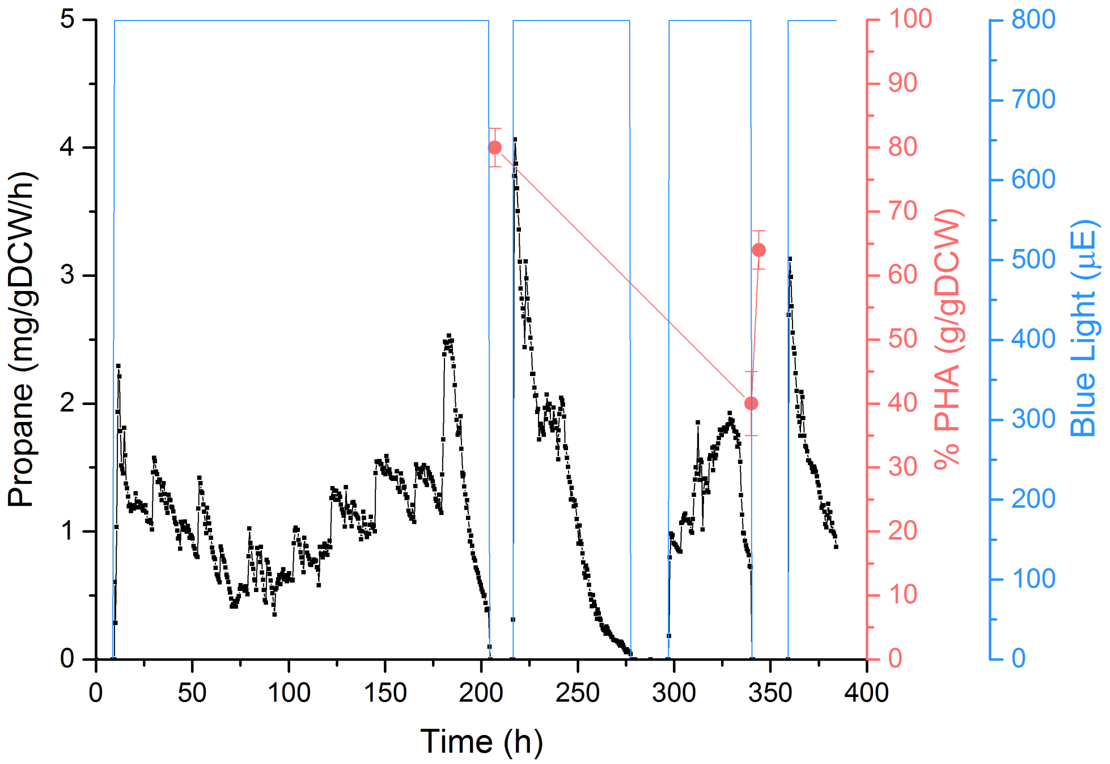

Figure S5. Fermentative propane and PHA production of *H. bluephagenesis* TD1.0 containing genomic integrated Por-FAP-HMAS$_{I219V}$. The culture (400 mL) was grown in LB60 pH 6.8 at 30 °C. Butyric acid (50 mM) was added 1 h after the OD 680 nm reached 0.8, with constant blue light illumination (800 µE; 8 h turned on)). Cultures were maintained with constant aeration (1.25 L/min) and temperature for 380 h. Butyric acid (~10 mmol) was added periodically, as indicated by the grey circles. Periodic harvesting of 60% of the culture and refilling with LB60 was performed with the blue light switched off. After 10 h dark culture growth, the blue light was switched on again. Online headspace gas analysis for propane production was performed using a Micro GC, while PHA estimations were performed offline using a GC.

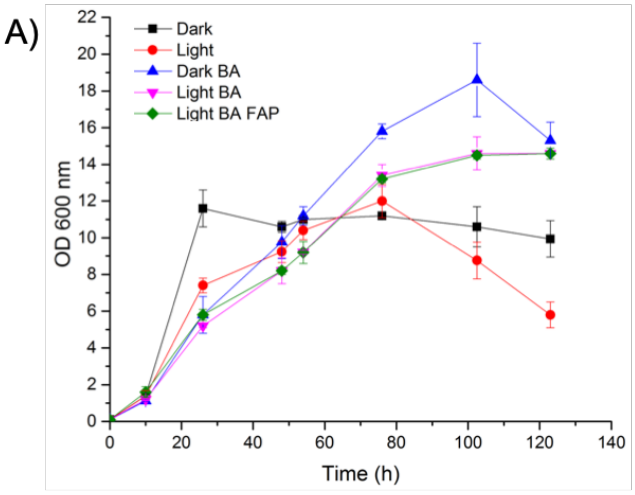

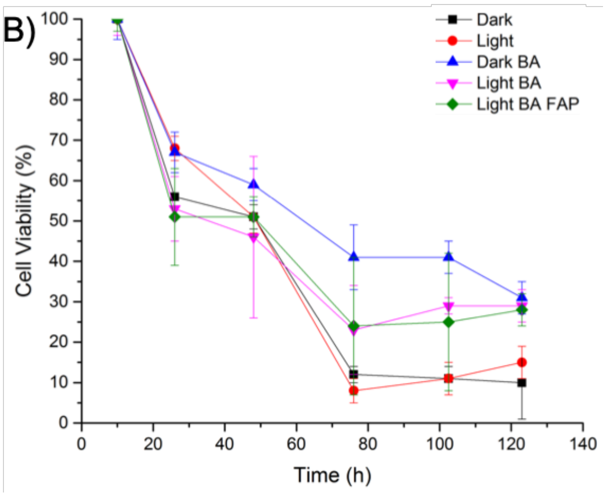

Figure S6. Effect of blue light and butyric acid on the A) growth and B) cell viability of *H. bluephagenesis* TD1.0 Por-FAP-HMAS$_{I219V}$. Cultures (100 mL) were grown in LB60 pH 6.8 at 30 °C in the presence or absence of butyric acid (50 mM) and blue light (800 µE). Cultures grown in the absence of blue light were maintained in the dark. Cell viability was determined using the CellTiter-Glo® 2.0 assay. Error bars represent one standard deviation of data from triplicate cultures. BA = butyric acid; FAP = culture contained a genome integrated Porin-FAP-HMAS$_{I219V}$ construct.

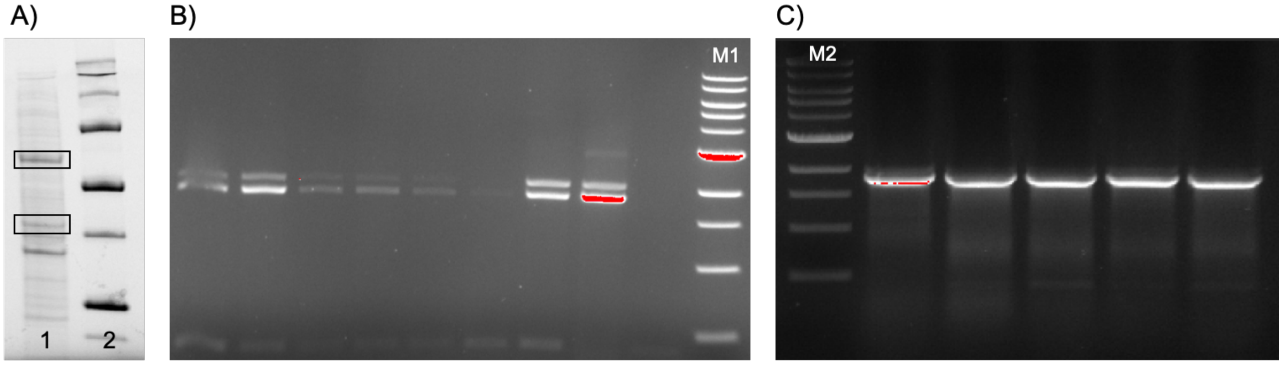

Figure S7. Qualitative analysis of CvFAP and HMAS expression and *hmgCAB* knockout in *H. bluephagenesis* TD1.0. A) SDS PAGE analysis of cell extracts of *H. bluephagenesis* Por-FAP-HMAS$_{I219V}$ with plasmid pHal2-HMAS$_{I219V}$. Lanes: 1 = cell extract; 2 = marker, with band sizes 250, 150, 100, 75, 50, 37, 25, 20 kD Protein bands CvFAP$_{G462V}$ (A) and SyHMAS$_{I219V}$ are indicated by black boxes. Expected sizes 66 kDa and 38 kDa. Gel electrophoresis of PCR reactions of individual *H. bluephagenesis* Por-FAP-HMAS$_{I219V}$ *HmgCAB*$^{-}$ knockout colonies B) before and C) after multiple rounds of re-streaking to single colonies. The expected band sizes for the *HmgCAB*$^{-}$ knockout and *HmgCAB* presence are 1771 and 2025 respectively. Molecular mass marker M1 has band sizes of 10, 8, 6, 5, 4, 3, 2, 1.5, 1, 0.5 kbp. Marker M2 has band sizes of 12, 10, 8, 6, 5, 4, 3, 2, 1.5, 1. 0.5 kbp.

A)

Parameter Estimates			
Factor	Estimate	t-Ratio	Probability (t)
Intercept	23.61 ± 2.89	8.15	< 0.0001 *
Temp	2.02 ± 3.02	0.67	0.5218
OD	5.83 ± 2.93	1.99	0.0824
Phe	-10.1 ± 3.14	-3.22	0.0123
Tyr	8.58 ± 2.93	2.93	0.0191
Gluc	4.32 ± 3.12	3.12	0.2061
**Effect Summary**			
Factor varied	LogWorth	P-Value	Significance
Phenylalanine	1.911	0.01228	*
Tyrosine	1.718	0.01913	*
OD 600 nm	1.084	0.08241	
Glucose	0.686	0.20611	
Temperature	0.283	0.52179	

B)

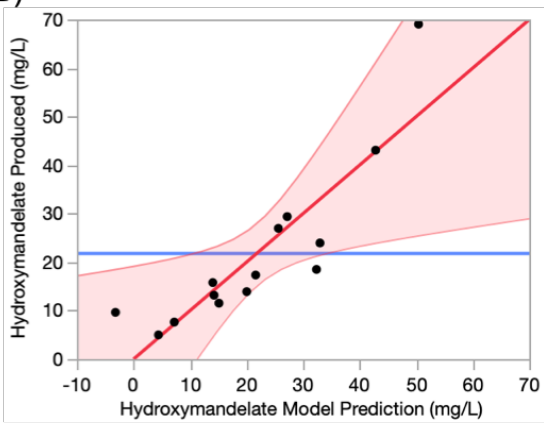

C)

D)

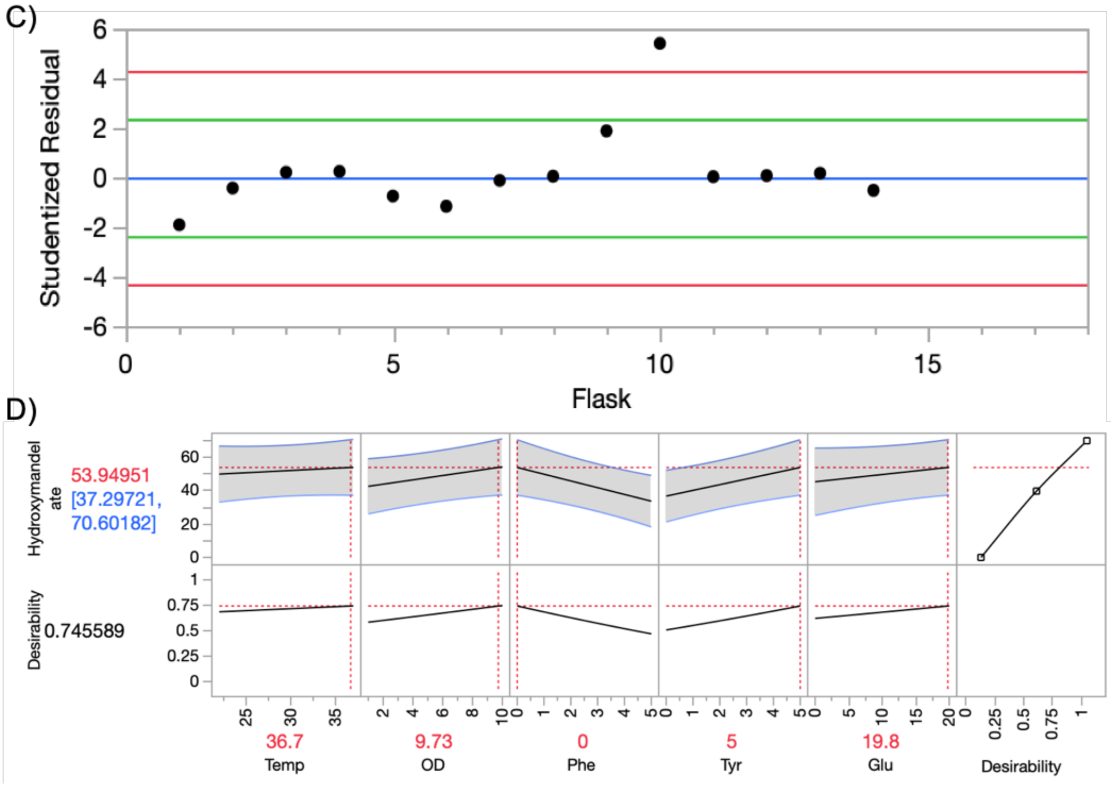

Figure S8. Summary of the first set of Design of Experiment (DOE) calculations for hydroxymandelate production. A) Table of the parameter estimations and the effect summary data. Impactful factors have a p-value of < 0.05 and are indicated by an asterisk. B) Actual verses predicted hydroxymandelate production plot from the JMP model. C) Studentized residuals plot for the data, showing there is one outlier data point. D) Prediction profile of the JMP model to assess optimal values for each factor to maximize hydroxymandelate production.

A)

Parameter Estimates			
Factor	Estimate	t-Ratio	Probability (t)
Intercept	21.64 ± 1.63	13.26	< 0.0001 *
Phe	-2.87 ± 1.80	-1.59	0.1348
Tyr	11.63 ± 1.80	6.47	< 0.0001*
Gluc	4.66 ± 1.80	2.59	0.0224*

Effect Summary			
Factor varied	LogWorth	P-Value	Significance
Phe	0.87	0.13484	
Tyr	4.675	0.00002	*
Gluc	1.649	0.02243	*

B)

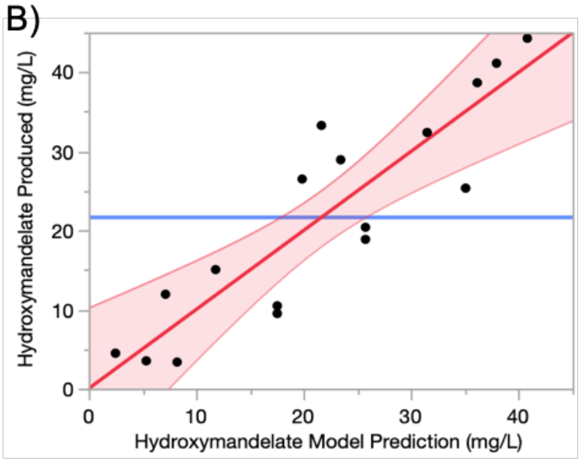

C)

D)

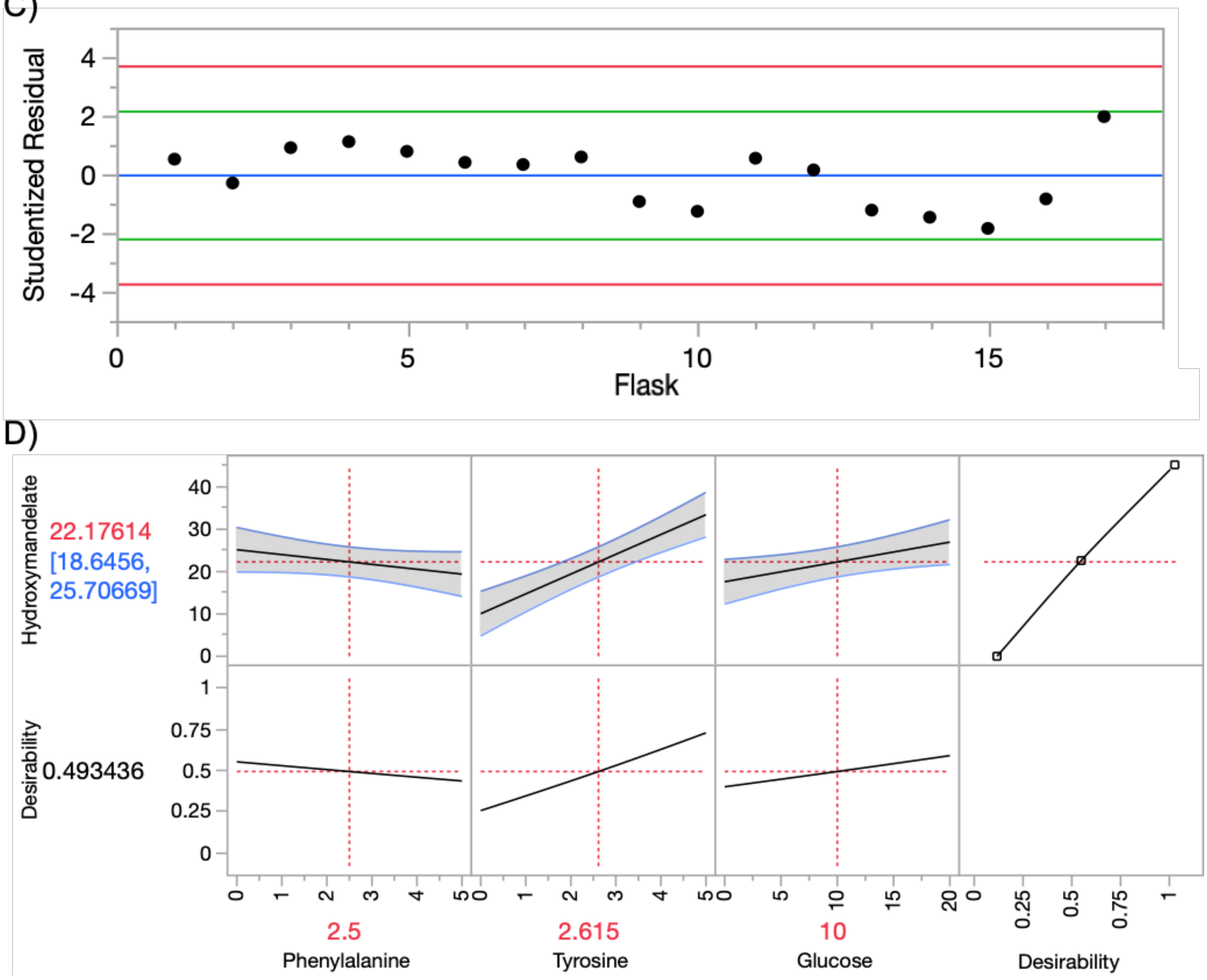

Figure S9. Summary of the second set of Design of Experiment (DOE) calculations for hydroxymandelate production. A) Table of the parameter estimations and the effect summary data. Impactful factors have a p-value of < 0.05 and are indicated by an asterisk. B) Actual verses predicted hydroxymandelate production plot from the JMP model. C) Studentized residuals plot for the data. D) Prediction profile of the JMP model to assess optimal values for each factor to maximize hydroxymandelate production.

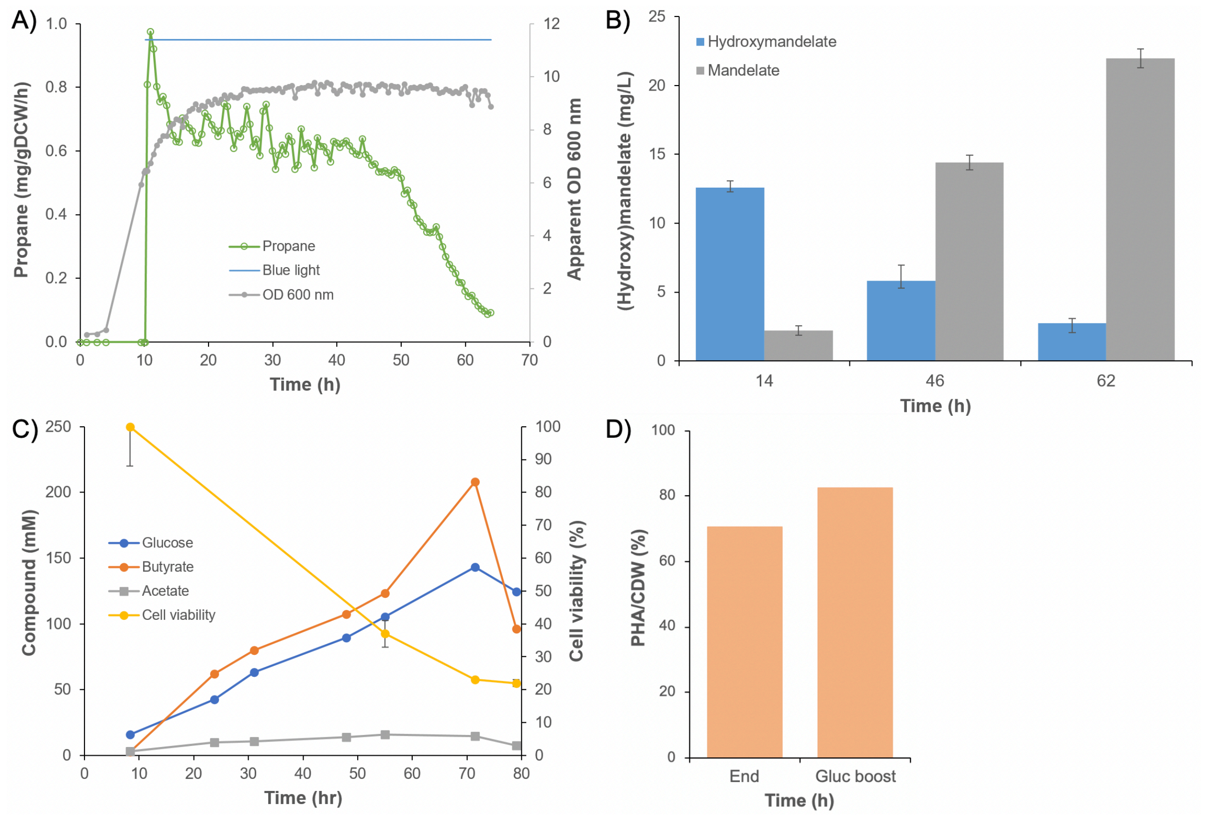

Figure S10. Fermentative production of propane, (hydroxy)mandelate and PHA by *H. bluephagenesis* Por-FAP-HMAS$_{I219V}$ *HmgCAB*$^{-}$ with pHal2-HMAS$_{WT}$ (Run 1). A) Culture growth of *H. bluephagenesis,* light induction, and propane production. B) (hydroxy)mandelate titer over time. C) Depletion of carbon sources and acetate production during fermentation of *H. bluephagenesis*, and cell viability over time. D) PHA at the end of fermentation, and three hours later after a glucose spike. The culture (400 mL) was grown in LB60 pH 6.8 at 30 °C with 1.25 L/min aeration until OD 600 nm reached 0.8, then butyric acid (25 mM) was added and the blue light (800 µE) was switched on 1 h later. When OD reached 4.6, 0.1M IPTG was added to induce HMAS plasmid. The culture was maintained for ~ 65 h with culture feeding of 4 mmol butyric acid, 4 mmol glucose, and 0.25 mmol Phe every 3 h. Offline analytics for metabolite monitoring were performed using HPLC and GC. Propane and culture optical density are shown as green and grey circles, respectively. The timing of the blue light is shown schematically as a blue line. Apparent OD 600 nm = photobioreactor optical density probe data corrected for non-linearity using the calibration curve in **Supplementary Figure S16**. Compound (mM) = glucose, butyrate or acetate concentration.

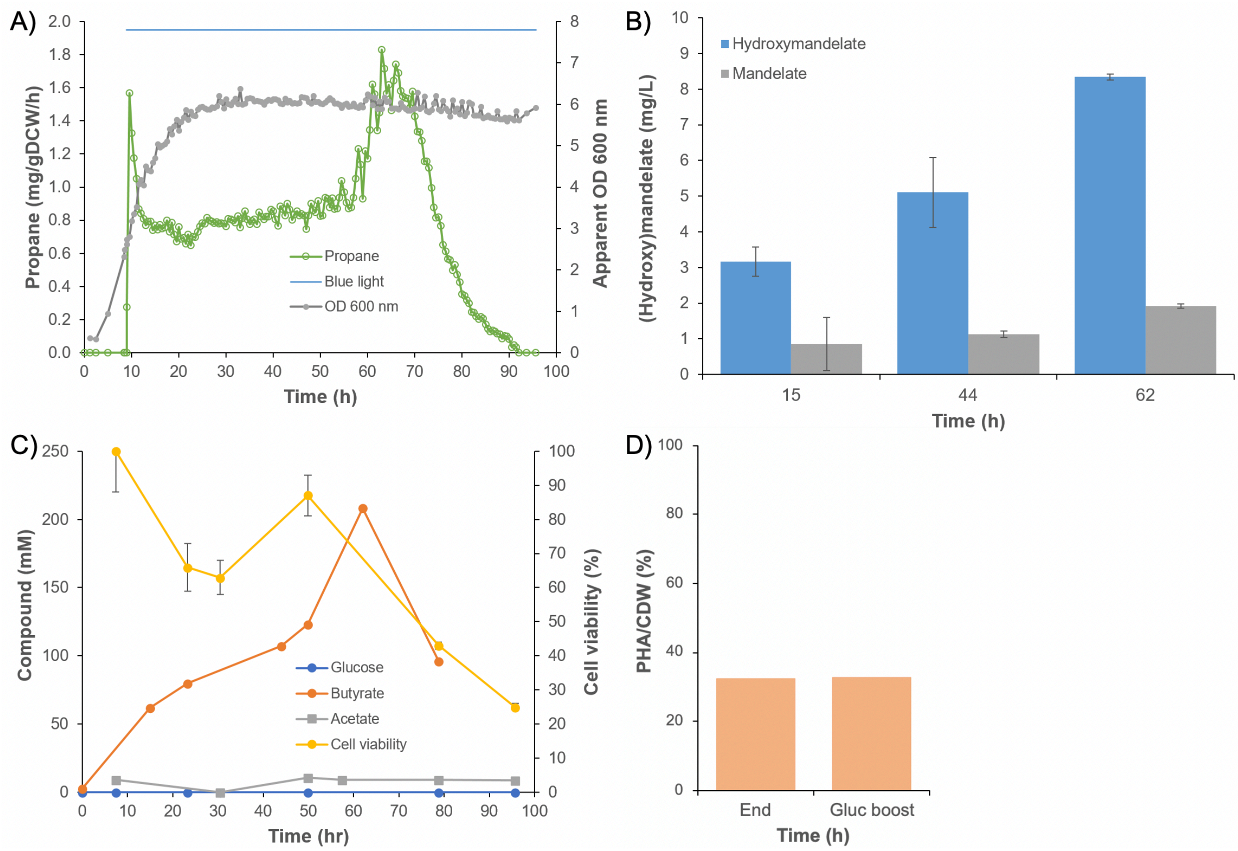

Figure S11. Fermentative production of propane, (hydroxy)mandelate and PHA by *H. bluephagenesis* Por-FAP-HMAS$_{I219V}$ *HmgCAB*$^{-}$ with pHal2-HMAS$_{I219V}$ (Run 2). A) Culture growth of *H. bluephagenesis,* light induction, and propane production. B) (hydroxy)mandelate titer over time. C) Depletion of carbon sources and acetate production during fermentation of *H. bluephagenesis*, and cell viability over time. D) PHA at the end of fermentation, and three hours later after a glucose spike. The culture (400 mL) was grown in LB60 pH 6.8 at 30 ºC with 0.3 L/min aeration until OD 600 nm reached 5.4, then butyric acid (25 mM) was added and the blue light (800 µE) was switched on 1 h later. 0.1M IPTG was added to induce plasmid. The culture was maintained for ~ 95 h with culture feeding of 8 mmol butyric acid and 0.25 mmol Tyr every 3 h. Offline analytics for metabolite monitoring were performed using HPLC and GC. Propane and culture optical density are shown as green and grey circles, respectively. The timing of the blue light is shown schematically as a blue line. Apparent OD 600 nm = photobioreactor optical density probe data corrected for non-linearity using the calibration curve in **Supplementary Figure S16**. Compound (mM) = glucose, butyrate or acetate concentration.

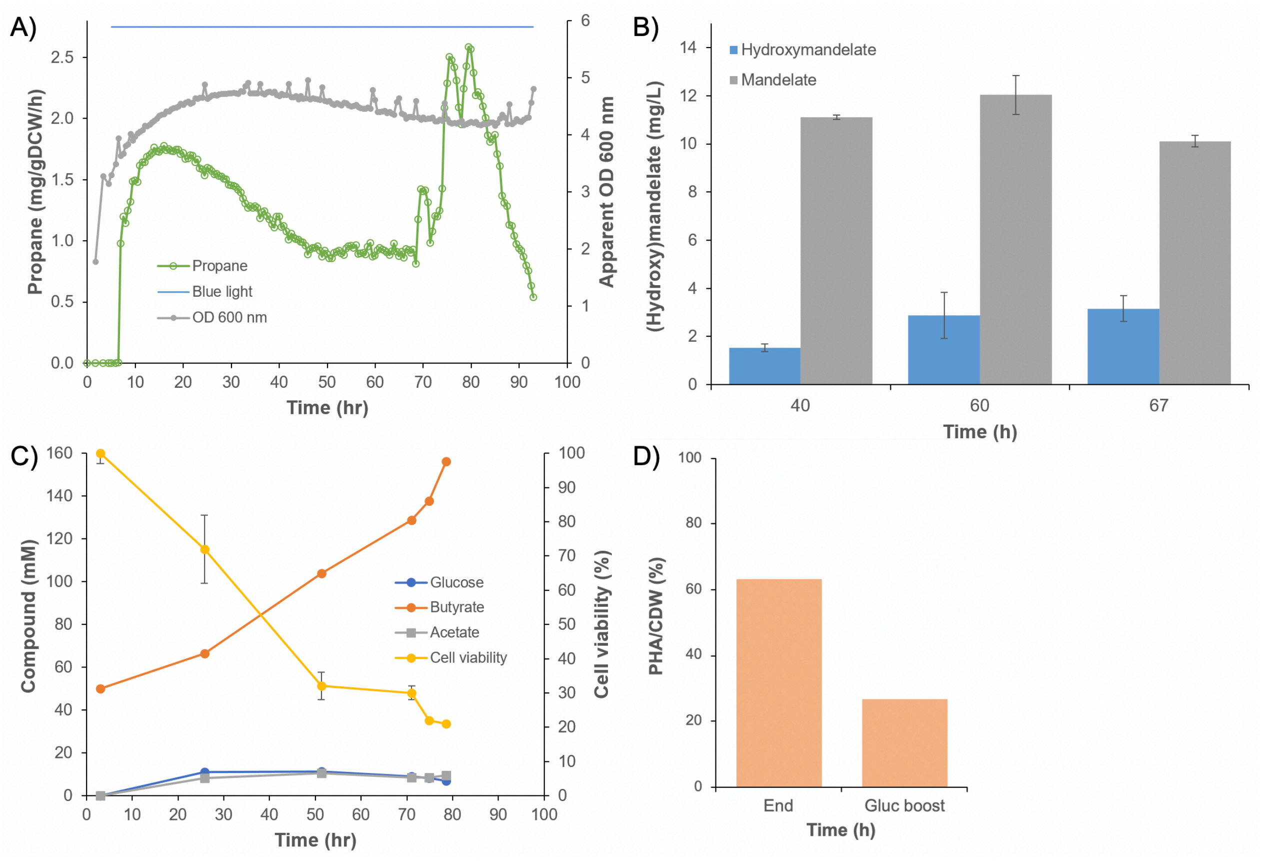

Figure S12. Fermentative production of propane, (hydroxy)mandelate and PHA by *H. bluephagenesis* Por-FAP-HMAS$_{I219V}$ *HmgCAB*$^{-}$ with pHal2-HMAS$_{I219V}$ (Run 3). A) Culture growth of *H. bluephagenesis* TD1.0, light induction, and propane production. B) (hydroxy)mandelate titer over time. C) Depletion of carbon sources and acetate production during fermentation of *H. bluephagenesis*, and cell viability over time. D) PHA at the end of fermentation, and three hours later after a glucose spike. The culture (400 mL) was grown in LB60 pH 6.8 at 30 °C with 0.48 L/min aeration until OD 600 nm reached 4.9. Then butyric acid (25 mM) was added, 0.1M IPTG was added with glucose (2.5 g/L) and amino acid (0.5 g/L each of Tyr and Phe) supplementation. The blue light (800 µE) was switched on 1 h later. The culture was maintained for ~ 95 h with culture feeding of 4 mmol butyric acid every 3 h. Offline analytics for metabolite monitoring were performed using HPLC and GC. Propane and culture optical density are shown as green and grey circles, respectively. The timing of the blue light is shown schematically as a blue line. Apparent OD 600 nm = photobioreactor optical density probe data corrected for non-linearity using the calibration curve in **Supplementary Figure S16**. Compound (mM) = glucose, butyrate or acetate concentration.

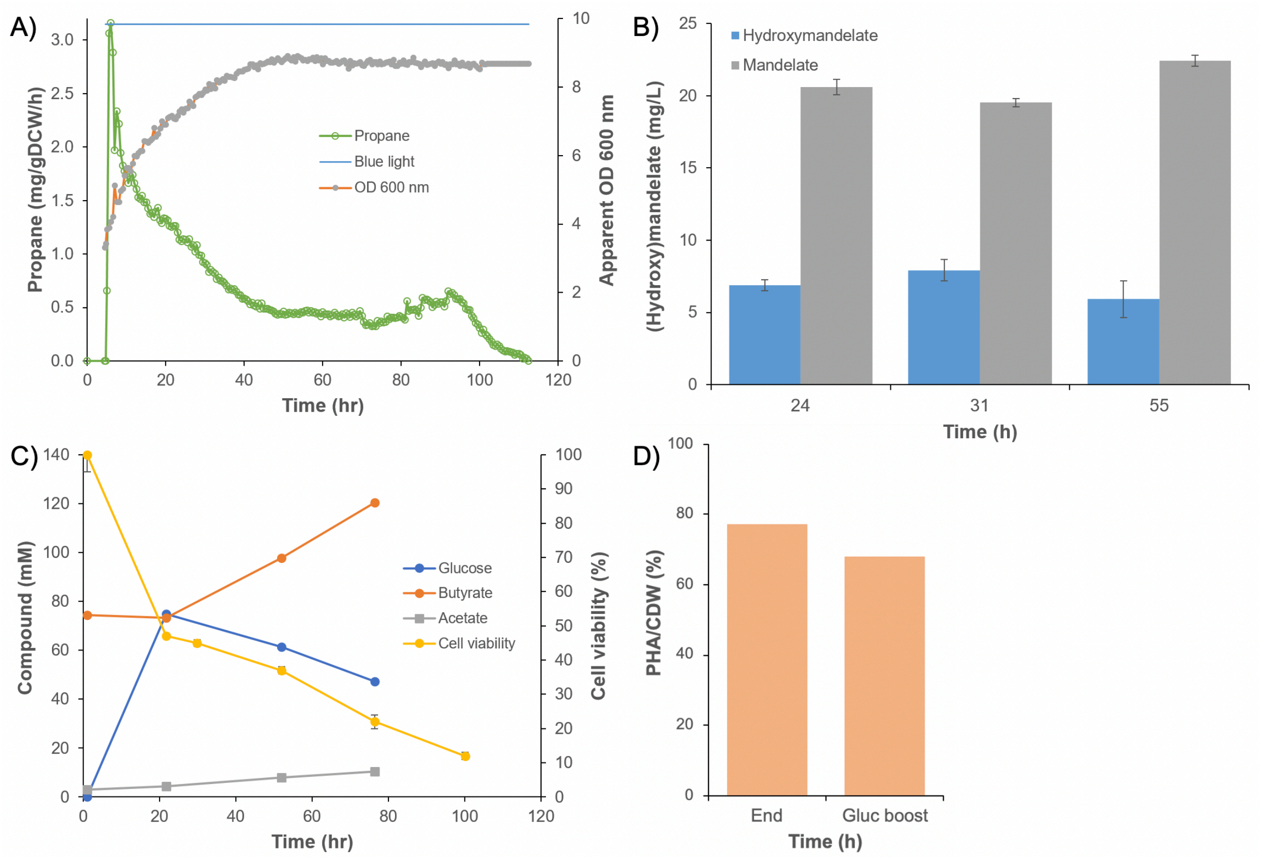

Figure S13. Fermentative production of propane, (hydroxy)mandelate and PHA by *H. bluephagenesis* Por-FAP-HMAS$_{I219V}$ *HmgCAB*$^{-}$ with pHal2-HMAS$_{I219V}$ (Run 4). A) Culture growth of *H. bluephagenesis* TD1.0*,* light induction, and propane production. B) (hydroxy)mandelate titer over time. C) Depletion of carbon sources and acetate production during fermentation of *H. bluephagenesis*, and cell viability over time. D) PHA at the end of fermentation, and three hours later after a glucose spike. The culture (400 mL) was grown in LB60 pH 6.8 at 30 °C with 1.25 L/min aeration until OD 600 nm reached 1, then butyric acid (25 mM) was added, and the blue light (800 μE) was switched on 1 h later. The culture was grown until OD 600 of 11, then 0.1 M IPTG was added with glucose (2.5 g/L) and amino acid (0.5 g/L each of Tyr and Phe) supplementation. The culture was maintained for ~ 115 h with culture feeding of 4 mmol butyric acid every 3 h. Offline analytics for metabolite monitoring were performed using HPLC and GC. Propane and culture optical density are shown as green and grey circles, respectively. The timing of the blue light is shown schematically as a blue line. Apparent OD 600 nm = photobioreactor optical density probe data corrected for non-linearity using the calibration curve in **Supplementary Figure S16**. Compound (mM) = glucose, butyrate or acetate concentration.

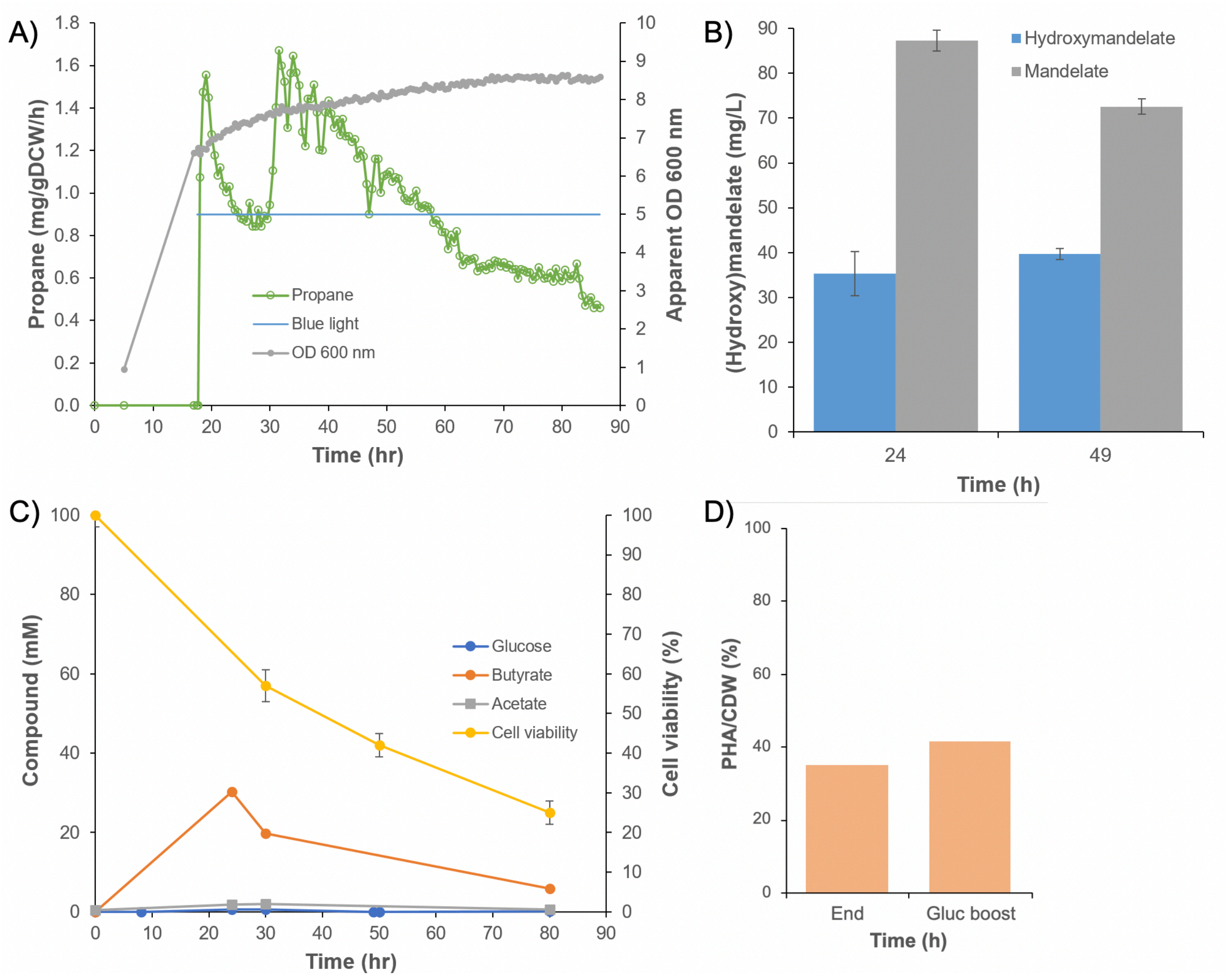

Figure S14. Fermentative production of propane, (hydroxy)mandelate and PHA by *H. bluephagenesis* Por-FAP-HMAS$_{I219V}$ *HmgCAB*$^{-}$ with pHal2-HMAS$_{I219V}$ (Run 5). A) Culture growth of *H. bluephagenesis* TD1.0, light induction, and propane production. B) (hydroxy)mandelate titer over time. C) Depletion of carbon sources and acetate production during fermentation of *H. bluephagenesis*, and cell viability over time. D) PHA at the end of fermentation, and three hours later after a glucose spike. The culture (400 mL) was grown in LB60 pH 6.8 at 30 ºC with 1.25 L/min aeration until OD 600 nm reached 5, then butyric acid (25 mM) was added, and the blue light (800 µE) was switched on 1 h later. Once OD reached 11, 0.1 M IPTG was added with glucose (2.5 g/L) and amino acid (0.5 g/L each of Tyr and Phe) supplementation. The culture was maintained for ~ 115 h with culture feeding of 2 mmol butyric acid every 3 h. Offline analytics for metabolite monitoring were performed using HPLC and GC. Propane and culture optical density are shown as green and grey circles, respectively. The timing of the blue light is shown schematically as a blue line. Apparent OD 600 nm = photobioreactor optical density probe data corrected for non-linearity using the calibration curve in **Supplementary Figure S16**. Compound (mM) = glucose, butyrate or acetate concentration.

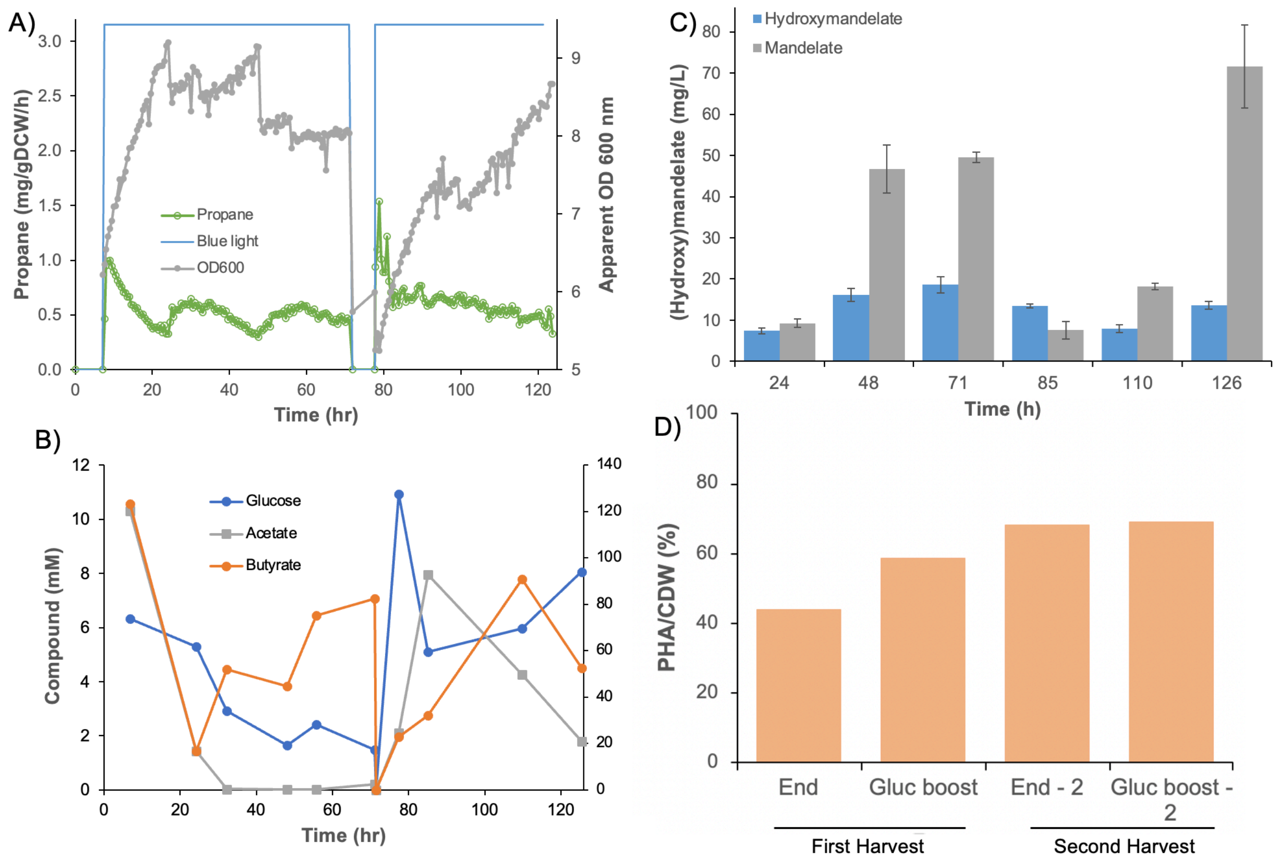

Figure S15. Fermentative production of propane, (hydroxy)mandelate and PHA by *H. bluephagenesis* Por-FAP-HMAS$_{I219V}$*HmgCAB*$^{-}$ with pHal2-HMAS$_{I219V}$. The culture (400 mL) was grown in LB60 pH 6.8 at 30 °C with 1.25 L/min aeration. The fermentation consisted of two batches with a dark harvest period at 70 and 126 hours. In the first and second batch after OD 600 nm reached 9 and 5.6 respectively, butyric acid (25 mM) was added and the blue light (400 µE) was switched on 1 h later. During harvest ~350 culture was removed for PHA harvesting, and fresh LB60, antibiotic, glucose, tyrosine and phenylalanine were added to allow culture to grow. A) Culture growth of *H. bluephagenesis*. B) Depletion of carbon sources and acetate production during fermentation of *H. bluephagenesis*. Between harvests the culture was maintained with butyric acid 25 mM twice daily. Offline analytics for metabolite monitoring were performed using HPLC and GC. Propane and culture optical density are shown as green and grey circles, respectively. The timing of the blue light is shown schematically as a blue line. Apparent OD 600 nm = photobioreactor optical density probe data corrected for non-linearity using the calibration curve in **Supplementary Figure S16**. Compound (mM) = glucose, butyrate or acetate concentration.

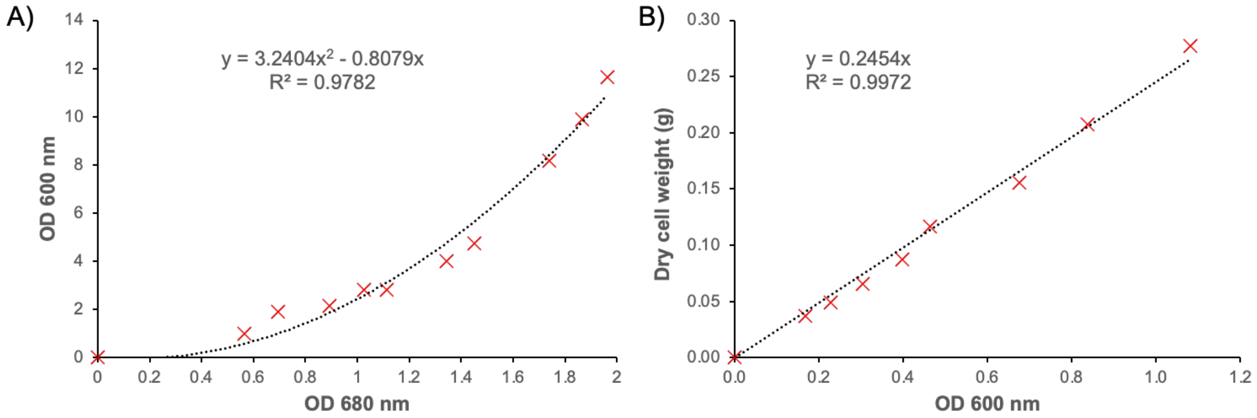

Figure S16. Calibration curves for the conversion of A) photobioreactor optical density probe (680 nm) to spectrophotometer OD 600 nm and B) OD 600 nm to dry cell weight of *H. bluephagenesis* 1.0. OD 680 nm probe readings were raw data from the photobioreactor online monitoring system. The OD 600 nm values were measured in a spectrophotometer after diluting *H. bluephagenesis* culture samples with water to be within the linear range (OD 600 nm < 1.0) and the values were adjusted for the dilution factor. Dry cell weights were obtained by diluting *H. bluephagenesis* cultures to the specified optical densities, followed by centrifugation to remove the culture supernatant. Cell pellets were freeze dried overnight and weighed to obtain the dry cell weights.

Supplementary Tables

Table S1. Expression constructs for *H. bluephagenesis* TD1.0 and genome integrated variants in this study.

Construct	Promoter-Gene(s)	Plasmid/Node	Ref.
Plasmid constructs			
pHal2-FAP$_{G462I}$	*T7L*-CvFAP$_{G462I}$	pHal2	(Amer *et al.*, 2020b)
pBbE2c-SyHMAS	*T7*-SyHMAS$_{WT}$	pBbE2c	(Robinson *et al.*, 2020)
pSEVA-h2	*T7*-SyHMAS$_{I219V}$	pSEVA341	(Robinson *et al.*, 2020)
pSEVA-HMO-HMAS	*T7*-ScHMO-SyHMAS$_{I219V/S204V}$	pSEVA341	(Robinson *et al.*, 2020)
pHal2-FAP-HMAS$_{I219V}$	*T7L*-CvFAP$_{G462I}$-SyHMAS$_{I219V}$	pHal2	This work
pHal2-HMAS$_{WT}$	*T7L*-SyHMAS$_{WT}$	pHal2	This work
pHal2-HMAS$_{I219V}$	*T7L*-SyHMAS$_{I219V}$	pHal2	This work
pHal2-HMAS$_{I219V/S204V}$	*T7L*-SyHMAS$_{I219V/S204V}$	pHal2	This work
pSBR1Ks-i-SceI	*trc*-I-SceI	pSceI	(Amer *et al.*, 2020a)
pSH-CvFAP$_{G462I}$	CvFAP$_{G462I}$	pSH[a]	(Amer *et al.*, 2020b)
pSH-FAP-HMAS$_{I219V}$	CvFAP$_{G462I}$-SyHMAS$_{I219V}$	pSH[a]	This work
pPor-FAP-HMAS$_{I219V}$	HA1-CvFAP$_{G462I}$-SyHMAS$_{I219V}$-HA2	pSH[a]	This work
pFumR-FAP-HMAS$_{I219V}$	HA1-CvFAP$_{G462I}$-SyHMAS$_{I219V}$-HA2	pSH[a]	This work
pCOG-FAP-HMAS$_{I219V}$	HA1-CvFAP$_{G462I}$-SyHMAS$_{I219V}$-HA2	pSH[a]	This work
pHal2-*tet*-hmgH1H2	hmgH1-tet-hmgH2	pHal2	This work
H. bluephagenesis TD1.0 genomic integrated constructs			
Por-FAP-HMAS$_{I219V}$	CvFAP$_{G462I}$-SyHMAS$_{I219V}$	Node *Por*[a]	This work
FumR-FAP-HMAS$_{I219V}$	CvFAP$_{G462I}$-SyHMAS$_{I219V}$	Node *FumR*[a]	This work
COG-FAP-HMAS$_{I219V}$	CvFAP$_{G462I}$-SyHMAS$_{I219V}$	Node *COB*[a]	This work
Por-FAP-HMAS$_{I219V}$ with HmgCAB$^-$ deletion	CvFAP$_{G462I}$-SyHMAS$_{I219V}$ Tet-R	Node *Por*[a] Node *HmgCAB*[b]	This work

[a]Suicide vector used for genomic integration of cassettes into *H. bluephagenesis* (Amer et al., 2020a, Amer et al., 2020b). [b]Tetracycline-resistance gene flanked by two homology arms of the start and end of the genomic encoded HmgC and HmgB genes, respectively. Tet-R = tetracycline resistance promoter and gene. Recombinant enzymes: FAP = CvFAP$_{G462I}$ variant; SyHMAS (WT and variants) = 4-hydroxymandelate synthase from *Streptomyces yokosukanensis*. *H. bluephagenesis* TD1.0 endogenous genes: Por = porin; FumR = fumarate reductase; COG = universal stress protein COG0589 and I-SceI = restriction endonuclease *Sce*I. Promoters are shown in italics and amino acid changes are shown as a subscript. *T7L* = *H. bluephagenesis* -compatible T7-like IPTG-inducible promoter (Zhao *et al.*, 2017). Plasmids pSEVA341 and pBbE2c are *E. coli* compatible, containing a T7 promoter. All plasmids have spectinomycin resistance, except for the pSH plasmids which have chloramphenicol resistance. All genome integrated constructs are chloramphenicol resistant, except for Por-FAP-HMAS$_{I219V}$ HmgCAB$^-$ which is tetracycline resistant.

Table S2. Oligonucleotide sequences for the assembly of plasmids used in this study.

**Stage**	**Oligonucleotide sequence 5' to 3'**	**Template DNA**
Assembly of pHal2-FAP-HMAS$_{I219V}$ by In-Fusion cloning		
pHal2-FAP$_{G462V}$ vector opening	TTATGCTGCAACGGTTGCCG<br>CACCGCTGAGCAATAAAGCCC	pHal2-FAP$_{G462V}$
HMAS$_{I219V}$ PCR	TATTGCTCAGCGGTGttagtgttctcttccggtgcc<br>ACCGTTGCAGCATAAGGAGGACAGCTAaatgcagtatgcgcatcgc	pSEVA-h2
Assembly of pHal2-HMAS wild-type and variants by In-Fusion cloning		
pHal2 with T7L vector opening	CACCGCTGAGCAATAAAGCCc<br>GGTATATCTCCTTCTTAAAGTTAAACAAActagtatt	pHal2-FAP
HMAS$_{I219V}$ PCR	TATTGCTCAGCGGTGttagtgttctcttccggtgcc<br>ACCGTTGCAGCATAAGGAGGACAGCTAaatgcagtatgcgcatcgc	pSEVA-h2
HMAS$_{WT}$ PCR	TATTGCTCAGCGGTGttagtgttctcttccggtgcc<br>ACCGTTGCAGCATAAGGAGGACAGCTAaatgcagtatgcgcatcgc	pSEVA-HMO-HMAS
HMAS$_{I219V,S204V}$ PCR	TATTGCTCAGCGGTGttagtgttctcttccggtgcc<br>ACCGTTGCAGCATAAGGAGGACAGCTAaatgcagtatgcgcatcgc	pBbE2c-HMAS
Assembly of pSH-FAP-HMAS$_{I219V}$ with chloramphenicol resistance using NEBuilder		
Vector opening between FAP and Chl^R genes	*CACCGCTGAGCAATAAAGCCc*<br>*TTATGCTGCAACGGTTGCCG*	pSH-CvFAP$_{G402I}$
HMAS$_{I219V}$ PCR	TATTGCTCAGCGGTGttagtgttctcttccggtgcc<br>ACCGTTGCAGCATAAGGAGGACAGCTAaatgcagtatgcgcatcgc	pHal2-FAP-HMAS$_{I219V}$
Assembly of pPor-FAP-HMAS$_{I219V}$, pFumR-FAP-HMAS$_{I219V}$ and *pCOG-FAP-HMAS$_{I219V}$ using NEBuilder*		
Replication Origin PCR[a]	GTACCGAGCTCGAGACGTAAAAAGGC<br>CAGTCTAGACTCGAGATTACCCTGTTATCCC	pHal2-FAP-HMAS$_{I219V}$
FAP-HMAS-Chl	gTTTGTTTAACTTTAAGAAGGAGATATACCATGGCCAG<br>gtaccgcatgcaatcatggtctatatgaata	pSH-FAP-HMAS$_{I219V}$
Por-HA1	TCTCGAGCTCGGTACTAATACTGTCAATCCGGCGCTCTAGCAG<br>gattgcatgcggtacAGCTTGGCTCAAGCCATGTTTAGCAGGT	TD1.0 genome
Por-HA2	AAACAAAcTTAGAAGTGGTAACGCGCGC<br>CTCGAGTCTAGACTGATGAAAAAGACACTTTTAGCGACTGCTATC ATTG	TD1.0 genome
COG-HA1	gaccatgattgcatgcggtacatgagctatcaccacatattagtagccgttgatc<br>CCTTTTTACGTCTCGAGCTCGGTACcggcggagagtccaaccgtctg	TD1.0 genome
COG-HA2	GGGATAACAGGGTAATCTCGAGTCTAGACTGtactatggcttcaacaattggttt tcgc<br>GGCCATGGTATATCTCCTTCTTAAAGTTAAACAAAcggctaatctccttattg ataattaacaaagaaaacg	TD1.0 genome
FumR-HA1	Gaccatgattgcatgcggtacggggagaaaagatcatgcataaatcatcatcg<br>CCTTTTTACGTCTCGAGCTCGGTACggtgtagccatgatggtggctttc	TD1.0 genome
FumR-HA2	GGCCATGGTATATCTCCTTCTTAAAGTTAAACAAActcacccttaaacagc gcaattaaggtatg<br>GGGATAACAGGGTAATCTCGAGTCTAGACTGgttgcgcgagcgctatgccaa aattc	TD1.0 genome
Assembly of pHal2-tet-resistance-hmgKO (pHal2-tet-hmgH1H2) using NEBuilder		
Tet^R PCR	GAAAAGTGCCACCTGACGTCTAAGAAAC<br>AGTTCTCCGCAAGAATTGATTGGCT	pBR322[b]
Origin replication	GTACCGAGCTCGAGACGTAAAAAGGC<br>CAGTCTAGACTCGAGATTACCCTGTTATCCC	pHal2-FAP-HMAS$_{I219V}$
Hmg-H1 PCR	GGATAACAGGGTAATCTCGAGTCTAGACTGccagagcaggggcgagggc<br>CCAATCAATTCTTGCGGAGAACTgctgatgcgttagcacgcgcc	TD1.0 genome
Hmg-H2 PCR	CCTTTTTACGTCTCGAGCTCGGTACggtgccggataccttatccgtc<br>AGACGTCAGGTGGCACTTTTCcggcctgaaggtgttctcgg	TD1.0 genome

[a]High-copy-number ColE1/pMB1/pBR322/pUC origin of replication and FAP-HMAS used for all three pSH plasmids and the HmgKO plasmid. [b]From New England Biolabs. HA = homology arm in the genome of TD1.0. 1, 2, are two homology arms. Chl^R = chloramphenicol resistance gene. Bases in uppercase are the homologous overlap regions and the lower-case bases correspond to the genome sequence.

Table S3. DOE parameters and hydroxymandelate production for the first screen with *H. bluephagenesis*.

	DOE parameters					Product titre
Culture no'	Temperature (°C)	Induction OD 600 nm	Glucose (g/L)	Tyrosine (g/L)	Phenylalanine (g/L)	Hydroxymandelate (mg/L)
1	30	10	20	5	5	18.49 ± 1.19
2	30	1	0	0	0	11.53 ± 0.08
3	37	1	20	2.5	5	15.78 ± 0.86
4	22	10	0	2.5	0	-
5	37	10	20	0	2.5	29.38 ± 1.01
6	22	1	0	5	2.5	13.91 ± 0.65
7	37	10	10	0	0	23.94 ± 0.92
8	22	1	10	5	5	13.17 ± 1.05
9	37	5.5	0	5	0	-
10	22	5.5	20	0	5	7.63 ± 0.51
11	37	1	0	0	5	9.64 ± 1.32
12	22	10	20	5	0	69.03 ± 3.65
13	37	1	20	5	0	43.06 ± 3.31
14	22	10	0	0	5	4.99 ± 0.63
15	37	10	0	5	5	26.96 ± 16.46
16	22	1	20	0	0	17.34 ± 15.51
17	30	5.5	10	2.5	2.5	43.45 ± 1.66

Cultures 4 and 9 failed to grow. Errors represent one standard deviation of the data from technical replicates.

Table S4. DOE parameters and hydroxymandelate production for the second screen with *H. bluephagenesis*.

	DOE parameters			Product titre
Culture no'	Glucose (g/L)	Tyrosine (g/L)	Phenylalanine (g/L)	Hydroxymandelate (mg/L)
1	0	0	5	41.12 ± 1.45
2	10	2.5	2.5	3.50 ± 0.63
3	20	2.5	5	28.92 ± 2.77
4	0	0	2.5	26.48 ± 2.56
5	20	5	0	11.92 ± 1.73
6	0	2.5	0	38.66 ± 2.81
7	20	0	0	4.47 ± 1.12
8	0	5	5	44.28 ± 3.77
9	0	5	0	20.38 ± 1.89
10	0	0	0	10.46 ± 1.41
11	20	0	5	15.04 ± 2.73
12	20	5	2.5	32.37 ± 2.71
13	10	0	5	18.86 ± 2.43
14	0	5	5	9.48 ± 2.03
15	10	5	0	25.33 ± 3.00
16	20	0	0	3.35 ± 0.60
17	20	5	5	33.26 ± 3.32

Errors represent one standard deviation of the data from technical replicates.

References

Amer, M., Hoeven, R., Kelly, P., Faulkner, M., Smith, M.H., Toogood, H.S., and Scrutton, N.S. (2020a) Renewable and tuneable bio-LPG blends derived from amino acids, *Biotechnol Biofuels* **13**: 125.

Amer, M., Wojcik, E.Z., Sun, C., Hoeven, R., Hughes, J.M.X., Faulkner, M., et al. (2020b) Low carbon strategies for sustainable bio-alkane gas production and renewable energy, *Energy Environ Sci* **13**: 1818-1831.

Fu, X.-Z., Tan, D., Aibaidula, G., Wu, Q., Chen, J.-C., and Chen, G.-Q. (2014) Development of *Halomonas* TD01 as a host for open production of chemicals, *Metab Eng* **23**: 78-91.

Gilman, J., Walls, L., Bandiera, L., and Menolascina, F. (2021) Statistical design of experiments for synthetic biology, *ACS Synth Biol* **10**: 1-18.

Qin, Q., Ling, C., Zhao, Y., Yang, T., Yin, J., Guo, Y., and Chen, G.Q. (2018) CRISPR/Cas9 editing genome of extremophile *Halomonas* spp, *Metab Eng* **47**: 219-229.

Robinson, C.J., Carbonell, P., Jervis, A.J., Yan, C., Hollywood, K.A., Dunstan, M.S., et al. (2020) Rapid prototyping of microbial production strains for the biomanufacture of potential materials monomers, *Metab Eng* **60**: 168-182.

Slaninova, E., Sedlacek, P., Mravec, F., Mullerova, L., Samek, O., Koller, M., et al. (2018) Light scattering on PHA granules protects bacterial cells against the harmful effects of UV radiation, *Appl Microbiol Biotechnol* **102**: 1923–1931.

Ye, J., Hu, D., Che, X., Jiang, X.-R., Li, T., Chen, J.-C., et al. (2018a) Engineering of *Halomonas bluephagenesis* for low cost production of poly(3-hydroxybutyrate-co-4-hydroxybutyrate) from glucose, *Metab Eng* **47**: 143-152.

Ye, J., Huang, W., Wang, D., Chen, F., Yin, J., Li, T., et al. (2018b) Pilot scale-up of poly(3-hydroxybutyrate-co-4-hydroxybutyrate) production by *Halomonas bluephagenesis* via cell growth adapted optimization process, *Biotechnol J* **13**: 1800074-1800010.

Zhao, H., Zhang, H.M., Chen, X., Li, T., Wu, Q., Ouyang, Q., and Chen, G.-Q. (2017) Novel T7-like expression systems used for *Halomonas*, *Metab Eng* **39**: 128-140.
